# Comparative genomic and transcriptomic analyses of transposable elements in polychaetous annelids highlight LTR retrotransposon diversity and evolution

**DOI:** 10.1186/s13100-021-00252-0

**Published:** 2021-10-29

**Authors:** Jonathan Filée, Sarah Farhat, Dominique Higuet, Laure Teysset, Dominique Marie, Camille Thomas-Bulle, Stephane Hourdez, Didier Jollivet, Eric Bonnivard

**Affiliations:** 1 Laboratoire Evolution, Genomes, Comportement, Ecologie CNRS, Université Paris-Sud, IRD, Université Paris-Saclay, Gif-sur-Yvette, France; 2 Marine Animal Disease Laboratory, School of Marine and Atmospheric Sciences, Stony Brook University, 100 Nicolls Road, Stony Brook, NY 11794-5000 USA; 3 Institut de Systématique, Evolution, Biodiversité (ISYEB) - Sorbonne Université, Muséum National d’Histoire Naturel, CNRS, EPHE, Université des Antilles, 7 quai Saint Bernard, 75252 Paris Cedex 05, France; 4 Sorbonne Université, CNRS, Institut de Biologie Paris-Seine, Laboratoire Biologie du Développement, UMR7622, “Transgenerational Epigenetics & small RNA Biology”, F-75005 Paris, France; 5 Sorbonne Université, CNRS, UMR 7144 AD2M, Station Biologique de Roscoff, Place Georges Teissier, 29688 Roscoff, France; 6 UMR8222 LECOB CNRS-Sorbonne Université, Observatoire Océanologique de Banyuls, 1 avenue Pierre Fabre, 66650 Banyuls-sur-Mer, France

**Keywords:** Transposable elements, LTR-retrotransposons, Polychaetous annelids, Transcriptoms, PIWI proteins

## Abstract

**Background:**

With the expansion of high throughput sequencing, we now have access to a larger number of genome-wide studies analyzing the Transposable elements (TEs) composition in a wide variety of organisms. However, genomic analyses often remain too limited in number and diversity of species investigated to study in depth the dynamics and evolutionary success of the different types of TEs among metazoans. Therefore, we chose to investigate the use of transcriptomes to describe the diversity of TEs in phylogenetically related species by conducting the first comparative analysis of TEs in two groups of polychaetes and evaluate the diversity of TEs that might impact genomic evolution as a result of their mobility.

**Results:**

We present a detailed analysis of TEs distribution in transcriptomes extracted from 15 polychaetes depending on the number of reads used during assembly, and also compare these results with additional TE scans on associated low-coverage genomes. We then characterized the clades defined by 1021 LTR-retrotransposon families identified in 26 species. Clade richness was highly dependent on the considered superfamily. Copia elements appear rare and are equally distributed in only three clades, GalEa, Hydra and CoMol. Among the eight BEL/Pao clades identified in annelids, two small clades within the Sailor lineage are new for science. We characterized 17 Gypsy clades of which only 4 are new; the C-clade largely dominates with a quarter of the families. Finally, all species also expressed for the majority two distinct transcripts encoding PIWI proteins, known to be involved in control of TEs mobilities.

**Conclusions:**

This study shows that the use of transcriptomes assembled from 40 million reads was sufficient to access to the diversity and proportion of the transposable elements compared to those obtained by low coverage sequencing. Among LTR-retrotransposons Gypsy elements were unequivocally dominant but results suggest that the number of Gypsy clades, although high, may be more limited than previously thought in metazoans. For BEL/Pao elements, the organization of clades within the Sailor lineage appears more difficult to establish clearly. The Copia elements remain rare and result from the evolutionary consistent success of the same three clades.

**Supplementary Information:**

The online version contains supplementary material available at 10.1186/s13100-021-00252-0.

## Background

Transposable elements (TEs) have been identified in all eukaryotic species investigated so far and can make up large fractions of genomes [1, 2]. They have a large impact on genome structure and stability, and are therefore considered to play an important role in evolution as one of the major sources of genetic variability in eukaryotes [3–5]. Environmental variations can promote genome plasticity through transcriptional activation and TEs mobilization, often in response to specific stimuli such as biotic stress and abiotic environmental changes [6–8]. TEs are very diverse in terms of structural features, sequences and replication mechanisms [9, 10]. They are classified into two classes [3, 9]: (i) DNA transposons (class II elements) replicate via a “cut and paste” mechanism. After transcription and translation, the autonomous elements produce the appropriate machinery for the recognition and transposition of a DNA intermediate, and (ii) retrotransposons (class I elements, specific to eukaryotes), replicate via a “copy and paste” mechanism, which relies on the reverse transcription of an RNA intermediate. Based on their mode of transposition, autonomous retrotransposons are subdivided into four major orders: (1) LTR (Long Terminal Repeats) retrotransposons, (2) LINEs (Long INterspersed Elements), also called non-LTR retrotransposons even if this term has no longer meaning because at least two other kinds of retrotransposons are also devoid of LTR: (3) Penelope and (4) YR (tyrosine recombinase encoding) elements [9]. Furthermore, on the basis of their structural features and phylogenetic relationships, these different categories can be divided into superfamilies. There are two groups among the Penelope elements, which could be considered as two superfamilies [11, 12], and three superfamilies have been characterised to date within LTR-retrotransposons [13]. Depending on the publication considered, few superfamilies of YR-retrotransposons are also distinguished [10, 14], and about twenty for both DNA transposons and LINEs [15–17]). These subdivisions can be found, more or less common, in the widely used transposable element libraries (e.g. RepeatMasker [18]) or databases (e.g. Repbase [19]). In addition to the autonomous elements that encode all the machinery necessary for transposition, non-coding elements can exist that can still be able to transpose by hijacking this machinery, such as MITEs for DNA transposons, SINEs for LINEs, or LARDs and TRIMs for LTR-retrotransposons.

In LTR-retrotransposons, the LTRs are composed of direct sequence repeats that flank the internal coding region. It is generally assumed that the mechanism of LTR retrotransposition is very similar among LTR-retrotransposons from divergent hosts. First, a retrotransposon RNA is transcribed by the cellularly-encoded RNA polymerase from a promoter located within the 5′ LTR to a termination site located within the 3′ LTR. The RNA is then translated in the cytoplasm to produce the proteins that form a virus-like particle (VLP) and carry out the reverse transcription and integration steps. LTR-retrotransposons usually encode two genes (*gag* and *pol*) in a single or two open reading frames [20]: the *gag* gene encodes structural proteins involved in the formation of the VLP; and the *pol* gene encodes several protein domains involved in the transposition mechanism, including a protease, an integrase, a reverse transcriptase (RT) and a RNaseH. These last two domains are always consecutive and adjacent. Therefore they are typically grouped into a single sequence (RT/RNaseH) that is conventionally used to reconstruct LTR-retrotransposon phylogenies [21].

Even if all LTR-retrotransposons remain quite similar in terms of structural features, sequences and replication mechanisms, they form distinct superfamilies (Copia, BEL/Pao and Gypsy) that can be subdivided into various clades based on the phylogeny of their most conserved domains. A TE clade refers to a monophyletic group of elements present in different host species, and more closely phylogenetically related clades may be grouped into a lineage. In the superfamily Copia, at least 11 clades have been distinguished in metazoans. Eight have only been reported from arthropods, more specifically in winged insects (Copia [22]), in Diptera (1731 and Xanthias [23]), or in a unique species (Tricopia, Mtanga, Humnum, Daphnia elements, Colesa1-like [13, 24–26]). The CoMol clade was only found in three species of mollusks [27]. Thus, only the GalEa and Hydra clades are described in multiple host phyla [13, 24]. The GalEa clade [28] is widely distributed among metazoans, whereas the detection of several Hydra elements in mollusks suggested that this clade may have a wider distribution than the few species in which it was previously described (a cnidarian, an amphipod and the zebrafish). In the BEL*/*Pao superfamily, elements have so far only been found in animals with nine clades currently described [27, 29, 30]. The two BEL and Pao clades were detected predominantly in insects, although two BEL elements were also reported in a sponge [29]. In fact, the original Pao clade has been divided into two separate clades Pao and Dan, the latter being represented in *Danio rerio* and some mollusks [27, 29]. The four clades Flow, Tas, Suzu and Sinbad have been observed in diverse phyla and it can thus be assumed that they are widely distributed in metazoans. By adding mollusk elements, the formerly recognized Sinbad clade was further divided into three distinct clades, which can be grouped together in the Sailor lineage [27]. The two new Bel/Pao clades, Sparrow and Surcouf, contain so far only elements from zebrafish and mollusks. The Gypsy superfamily is clearly the most diverse one with 34 clades listed in the GypsyDatabase [31], half of which observed in metazoans. In addition to the clades in this database two more clades have been reported in decapods and 16 in mollusks [24, 27]. While most clades have a fairly limited distribution (insects, vertebrates, nematodes or tunicates), five clades are present in several phyla: Gmr1, CsRN1 and the three A-clade, B-clade, C-clade that formed the most widespread Mag lineage.

The dynamics and evolutionary success of TEs among genomes are highly variable. Different measures can be used to quantify the abundance of TEs in a genome as the number of copies, the proportion of TEs, the number of different families or clades, or their host’s distribution (i.e. repartition of each family, clade or superfamily among different host species). In Opisthokonta, the three LTR-retrotransposon superfamilies display uneven relative abundances. Previous large-scale comparative analyses of various phyla (insect [29], crustaceans [24], fungi [32], Pezizomycotina [21], mollusks [27]) revealed that Gypsy and Copia elements display opposite distribution, representativeness and diversity. Gypsy elements are clearly the most abundant and highly diversified superfamily, with large numbers of copies and many families and clades. The Copia elements appear much rarer, absent in a third of the examined species, and generally exhibit a low number of copies and a low diversity in terms of both families and clades. These patterns suggest that Copia and Gypsy retrotransposons likely display two different dynamics. Gypsy elements could simply follow a Red Queen dynamics [33] in which elements constantly transpose and evolve to escape the host’s regulatory mechanisms. Conversely, even if they are affected by “arms race”, Copia elements, have a dynamics that mostly follow a “Domino Day spreading” model [21, 24, 27] in which only few clades are maintained due to amplification bursts in specific taxonomic groups. Finally, the BEL/Pao superfamily appears to be the second most abundant superfamily in terms of copy number, number of families and clade diversity [23, 27, 29]. The presence of BEL/Pao elements appears to be phylum-dependent as different patterns can be observed in different taxa. They are found in a majority of chordates, insects and nematodes, but have only been detected in half of the mollusks studied so far and seem to be absent in mammals. Phylum-dependency seems even more pronounced when the different BEL/Pao clades are considered [27]. Because of their intermediate patterns of distribution and diversity, the dynamics of the BEL/Pao elements is trickier to infer.

Recently, several eukaryotic taxa of ecological and evolutionary significance began to be investigated for the presence of TEs, but many of them still have received little attention. The precision provided by the reworking of the clades shows that the study of new host phyla can substantially improve the knowledge of TEs diversity and their evolution. In this context, annelids represent an important model to investigate transposable elements diversity within a phylum. With more than 18,000 living species, they display a large diversity of species inhabiting a wide range of environments depending on their group as earthworms in terrestrial, leeches in marine and freshwater, and polychaetes mainly in brackish, estuarine or marine ecosystems. Many polychaetes are errant, some are sedentary (living in tubes), and others are parasitic or commensal with bivalves or echinoderms. Despite their incredible diversity, annelids have received very little attention in the past regarding transposable elements (see Additional file 1 for a summary). Only few assembled genomes of polychaete are available. In *Capitella teleta* [34], the number of TE copies includes 57% of LINEs (mainly Proto and Crack), 12% of LTR-retrotransposons (Gypsy) and 10% of DNA transposons (mainly TC1/mariner and Maverick). In *Lamellibrachia luymesi* [35], the opposite is observed; DNA transposons are as frequent (42%) as the LINEs (40%), whereas the LTR-retrotransposons are rare (7%), with 182 intact copies, of which a large majority were Gypsy (178) and two Copia and two Bel/Pao (2) [36].

Studies involving several types of TEs are most often carried out either on one or few complete assembled genomes, or in particular phyla for which many genomes are available (such as insects or vertebrates). However, for the majority of phyla, the number of partial or complete genomes remains very limited, often too limited to give full access to the diversity and distribution of TEs between species. The lack of genomic resources can present challenges for studying TEs in non-model organisms. Fortunately, low-coverage NGS sequencing analyses using assembly-free software such as DNApipeTE [37] or RepARK [38], allow the detection and estimation of the proportion of TEs at reasonable cost. In addition, transcriptome sequencing offers an attractive method for gathering information about the diversity of TEs, especially in animals with large genomes. Transcriptome scans have already been used to occasionally characterize some new elements [39, 40] and the comparative description of ten transcriptomes of invertebrates inventoried TEs for the most redundant hits [41]. For example, our earlier study of mollusk LTR-retrotransposons clearly demonstrated the usefulness of using a combination of transcriptomic and genomic data in order to analyze transposable element diversity, in particular by improving or enabling the characterization of particular clades [27]. For non-model organisms with limited genomic information, this combination provides a cost-saving tool giving direct access to transcribed elements, therefore potentially autonomous and active; and to examine species with very large genomes. As genomic data for polychaetes are currently very scarce, we carried out low-coverage genomic sequencing for 14 polychaetes in addition to 26 transcriptomes. Our goal is to perform comparative genomics and transcriptomics of TEs within divergent groups of annelids with good coverage. For this purpose, we targeted two very distinct groups of polychaetes: Canalipalpata and Aciculata (=Errantia). For the former, our dataset includes three families: Ampharetidae, Terebellidae, and Alvinellidae. For the latter, our sampling encompasses diverse lineages of Polynoidae, a family that comprises over 900 species. Thus, we took advantage of these original data to carry out the first wide-scale comparative analysis of TEs in annelids. Our analyses show that the composition and abundance of TEs are highly variable in polychaete genomes but LTR elements dominate in most species. We thoroughly investigate the phylogenetic relationships between the different clades of LTR-retrotransposons and their distribution in this phylum, and compare results obtained by the two sequencing approaches to assess the rate of TEs representativeness in transcriptomes.

## Results

### Transposable elements in annelid genomes

**Fig. 1:**
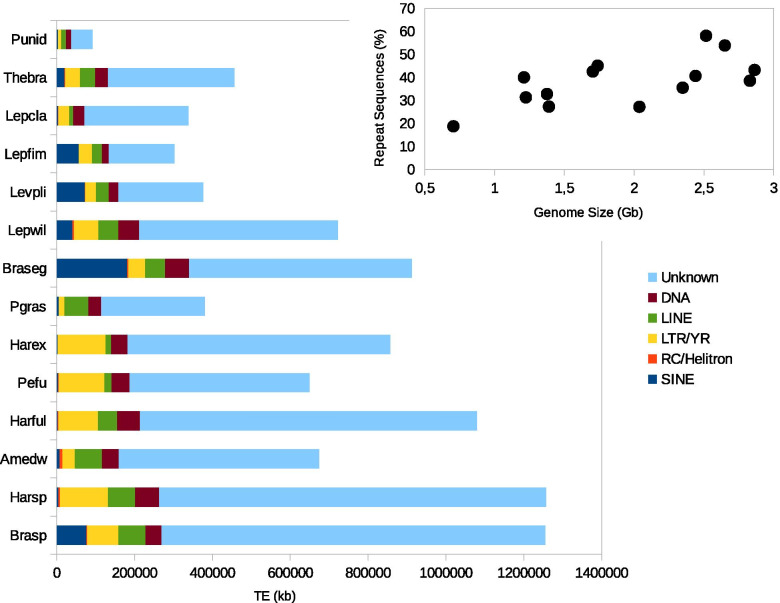
TEs proportions in 14 Annelid genomes. The global TEs content of each genome (%) have been plotted against the genome size in Gb (right) and the superfamily composition of the mobilome have been plotted for each species (left). Punid - *P. unidentata*; Thebra - *Thermopolynoe branchiata*; Lepcla - *Lepidonotus clava*; Lepfim - *Lepidonotopodium fimbriatum*; Levpli - *Levensteiniella plicata*; Lepwil - *Lepidonotopodium williamsae*; Braseg – *Branchinotogluma segonzaci*; Pgras - *P. grasslei*; Harex - *Harmothoe extenuata*; Pefu - *Pettitbonesia furcosetosa*; Harful - *Harmothoe fuligineum*; Amedw - *Neoamphitrite edwardsii*; Harsp - *Harmothoe* sp*.*; Brasp - *Branchinotogluma* sp*.*

Polychaete species used in this study belong to two distinct orders of Annelida (Additional file 2): The Phyllodocida (15 species of Polynoidae) and the Terebellida (6 species of Alvinellidae, 1 species of Ampharetidae and 4 species of Terebellidae). We first provided an estimation of the genome size of 13 species using flow cytometry (Additional file 3). Genome size is diverse, ranging from 700 Mb (*Paralvinella unidentata)* to 3.7Gb (*Thelepus* sp*.*) and some closely related species display large variation as for the genus *Lepidonotopodium* (1.2Gb and 2.3Gb). Quantitative analysis of the TEs content of each species revealed as expected a linear relationship between the genome size and the abundance of repeated elements (Fig. 1 right). Repeated elements account for more than 50% of the global contents of some large genomes such as *Branchinotogluma* sp*.* and *Harmothoe* sp*.* By contrast, the contribution of repeated elements is moderate for the smaller genomes (less than 20% for *P. unidentata*)*.* We also analyzed the TE superfamily composition of each genome by comparing the consensus sequences of each repeated family against Repbase (Fig. 1 left). Most of the repeated families do not match with any known TE families, up to over 75% in some genomes. This result is mainly explained by the scarcity of TEs identified so far in annelids. Repbase indeed includes only 200 annotated TE families (out of a total of 51,000) coming almost exclusively from the *C. telata* genome. These very small numbers of elements in Repbase limit considerably our ability to assign properly our sequences. Regarding the annotated orders, SINE elements are especially numerous in *Branchinotogluma segonzaci* genome, and LINE elements dominate the mobilome of *Paralvinella grasslei.* This seems to be isolated cases as for all of the other species, DNA and LINE elements account for a relatively small but constant proportion of the genome (5–10%). LTR elements are the most abundant superfamilies in most of the genomes (10–20% of the total TEs contents). We thus decided to focus our analysis on LTR elements in order to understand the diversity and evolution of these superfamilies in annelids.

### Transposable elements in annelid transcriptomes

**Fig. 2:**
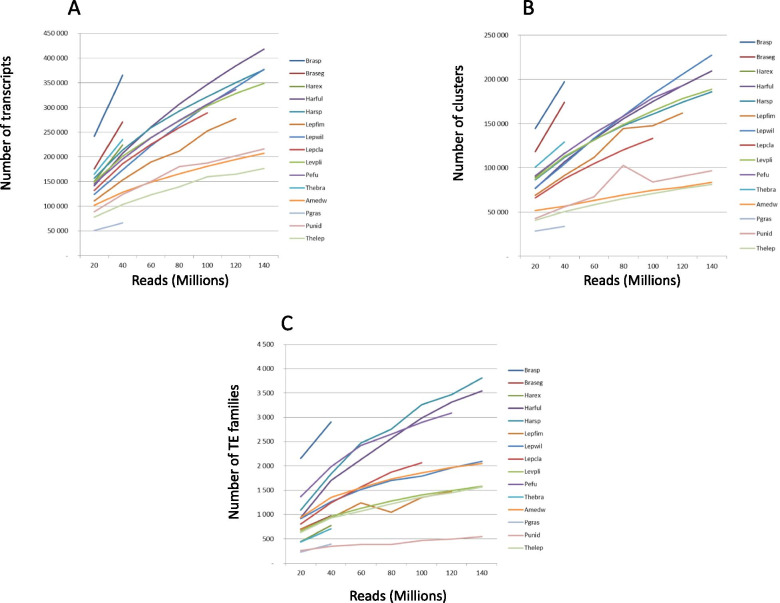
Assembly metrics for annelid transcriptomes. Assorted size metrics showing (**A**) Number of transcripts; (**B**) Number of clusters; (**C**) Number of TE families

Transcriptome analysis based on RNAseq datasets represents another tool to describe the diversity of active TEs without the need for reference genomes. But, as differences in expression levels may impact the coverage of any given sequence over several orders of magnitude, it remains unclear what is the ideal sequencing depth for our purpose. In order to study the influence of the sequencing effort on TEs detection, we computationally sub-sampled the original libraries into several sub-libraries, at regular increments of reads from 20 to 140 million, for which we compare trends from de novo assemblies using Trinity across 15 different annelid RNAseq (Fig. 2). The number of transcripts increases steadily with sequencing effort for all species (Fig. 2A). It varies greatly between species (from 60,000 to 350,000 sequences at 40 million reads per species), and seems quite high compared to what could be expected given the number of coding sequences in the genome, as previously described in various invertebrates [42]. The same pattern of increase is observed for Trinity clusters (Fig. 2B, each cluster represents the full transcriptional complexity for a given gene or set of genes that share sequences) as there is a good co-variation between clusters and transcripts regardless of the number of reads used (*R*^2^ = 0.92, Additional file 4). This indicates that, on average, the number of transcripts per cluster varies little. In contrast, for transposable elements, most curves approach a plateau for family counts (Fig. 2C). We define a TE family (an element) as a cluster of related TE copies within a given genome. The number of TE families increases sharply between 20 and 40 million reads and tends to level off after 40 million reads. It depends mainly on the species considered and the co-variations observed are less strong between the TEs diversity and either the number of transcripts or the number of clusters (R^2^ = 0.58 and R^2^ = 0.45, respectively, Additional file 4). Even if the maximum number of TEs does not seem to exceed 2000 families for most species, four species could potentially have more than 3000. Conversely, the TEs diversity appears to be very low in the alvinellid worms (*P. unidentata* (Punid) and *P. grasslei* (Pgras)), barely exceeding 500 TE families.

**Fig. 3:**
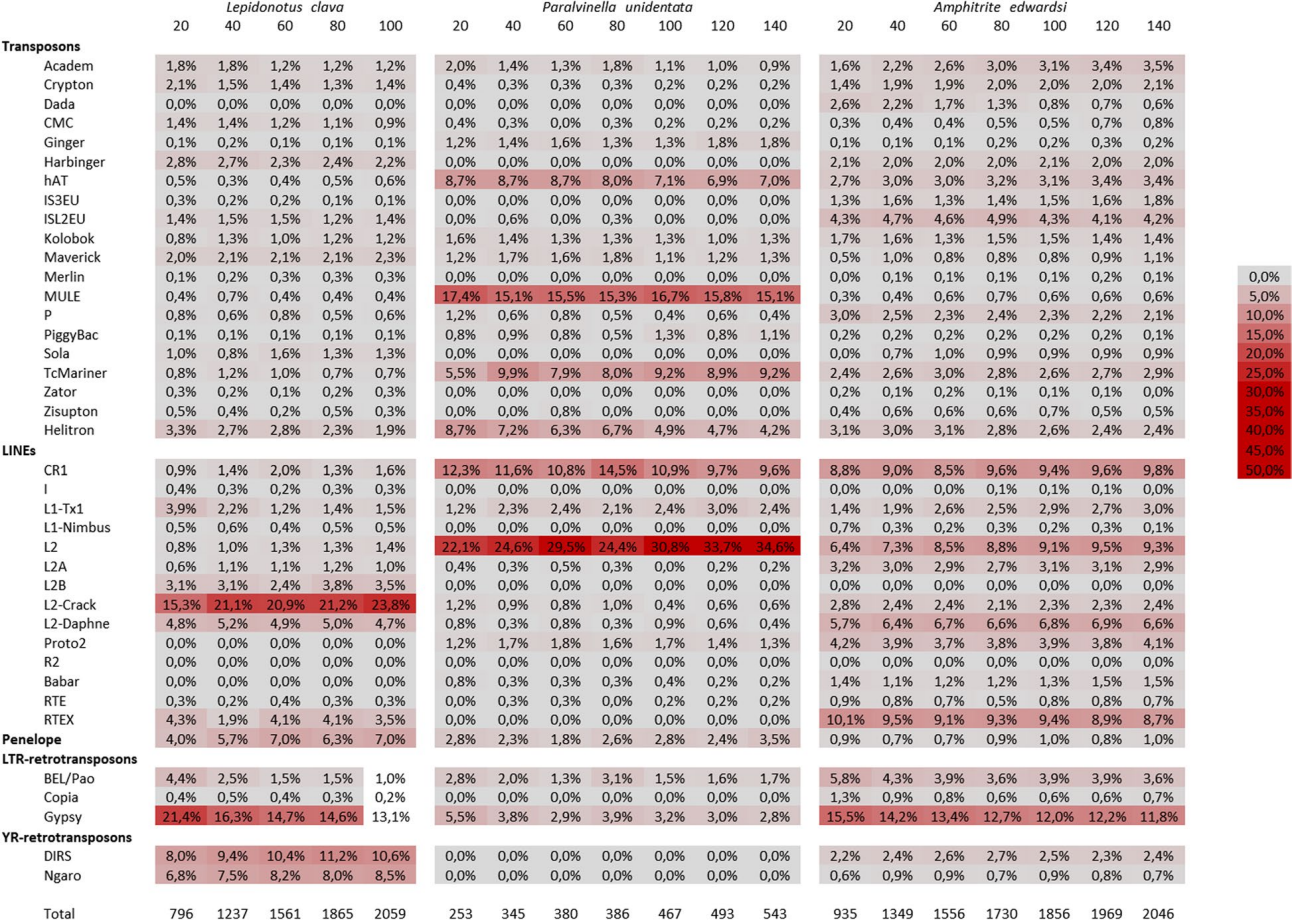
Heat maps of TE types identified in three annelids depending on the number of reads. Proportions of TEs are displayed as a color spectrum ranging from grey (low) to bright red (high) as shown in the legend. The columns are ordered according to the number of reads (indicated above in millions of reads) used to assemble the transcriptome

We investigated whether this increase in the number of families could affect the diversity in TEs by looking at the relative proportion of different element types obtained for distinct assemblies (Fig. 3, Additional file 5). For all the distribution profiles of TE types, there is no or very little variation between the different transcriptomes regardless of the number of reads used for the assemblies. The only notable differences mainly regard the transcriptomes assembled with the smallest number reads (20 million), where differences in the proportions of L1-Tx1, L2-crack, BEL/Pao and Gypsy elements or TcMariner elements can be perceived in *Lepidonotus clava* and *P. unidentata,* respectively. Other quantitative variations of elements were rare and punctual such as the decrease of RTEX elements in *L. clava* at 40 millions of reads (1.9% vs 4%).

**Fig. 4:**
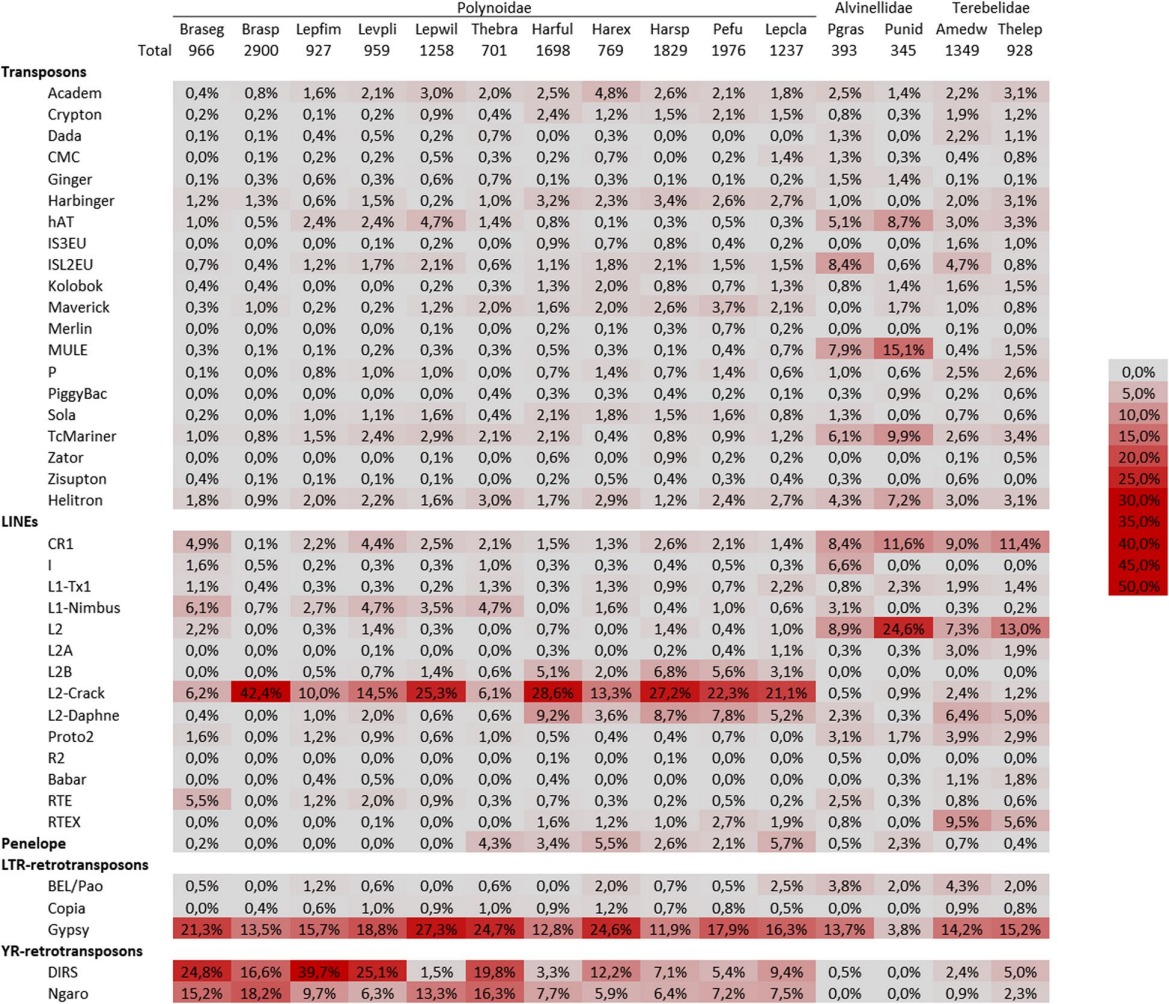
Heat map of TE types identified in 15 annelid species. Element proportions in the transcriptomes assembled from 40 million reads are shown as in Fig. 3. The total number of TE families detected is indicated above each column. The columns are ordered according to the probable phylogenetic relationships of species: Braseg – *Branchinotogluma segonzaci*; Brasp - *Branchinotogluma* sp*.*; Lepfim - *Lepidonotopodium fimbriatum*; Levpli - *Levensteiniella plicata*; Lepwil - *Lepidonotopodium williamsae*; Thebra - *Thermopolynoe branchiata*, Harful - *Harmothoe fuligineum*; Harex - *Harmothoe extenuata*; Harsp - *Harmothoe* sp*.*; Pefu - *Pettitbonesia furcosetosa*; Lepcla - *Lepidonotus clava*; Pgras - *P. grasslei*; Punid - *P. unidentata*; Amedw - *Neoamphitrite edwardsii*, Thelep - *Thelepus* sp*.*

We therefore decided to compare TEs abundances between species based on the profiles obtained with the 40 million reads assemblies (Fig. 4). The total number of TE families is quite low in the two Alvinellidae species (345 for *P. unidentata* and 393 for *P. grasslei*), much higher in the two Terebellidae (928 for *Thelepus* sp. and 1349 for *Amphitrite edwardsii*) and appears high but very variable in the eleven Polynoidae (between 701 for *Thermopolynoe branchiata* and 2900 for *Branchinotogluma* sp*.*). Considering the types of annelid TEs, the DNA transposons and Penelope elements are quite rare; LINEs and YR-retrotransposons are well represented and can take a very important part of the families in some species. Finally, a great diversity of Gypsy retrotransposon is detected in almost all species (between 12 and 27%, except in *P. unidentata*). Interestingly, quite different results are observed with a clear effect of the phylogeny of the hosts resulting in only three distribution patterns. (1) The Polynoidae are distinguished by a large amount of L2-Crack (6 to 42%) accompanied rather by L1-Nimbus for six species of the species or L2-Daphne for the other five (these two sets forming two distinct groups in the classification of species, Additional file 2), and a large number of Ngaro (6 to 18%) and DIRS (5 to 40% in *Lepidonotopodium fimbriatum* with the exception of *L. williamsae* and *Harmothoe fuligineum*). (2) In Alvinellidae, the DNA transposons hAT, ISL2EU, MULE, TcMariner and Helitron are well represented (4 to 15%); CR1 and L2 are the most frequent among LINEs; the BEL/Pao families are quite numerous (more than 2%); and there are no or very few families of Copia or YR-retrotransposons. (3) In Terebellidae, the DNA transposons seem to be rather poorly diversified (even if they are perhaps a little more diverse than in Polynoidae); as for the Alvinellidae there is an important diversity of CR1 and L2, but also of L2-Daphne and RTEX; the BEL/Pao and DIRS are quite diverse, which is less the case for the Ngaro.

### Comparison of TEs obtained from genomic or transcriptomic data

**Fig. 5:**
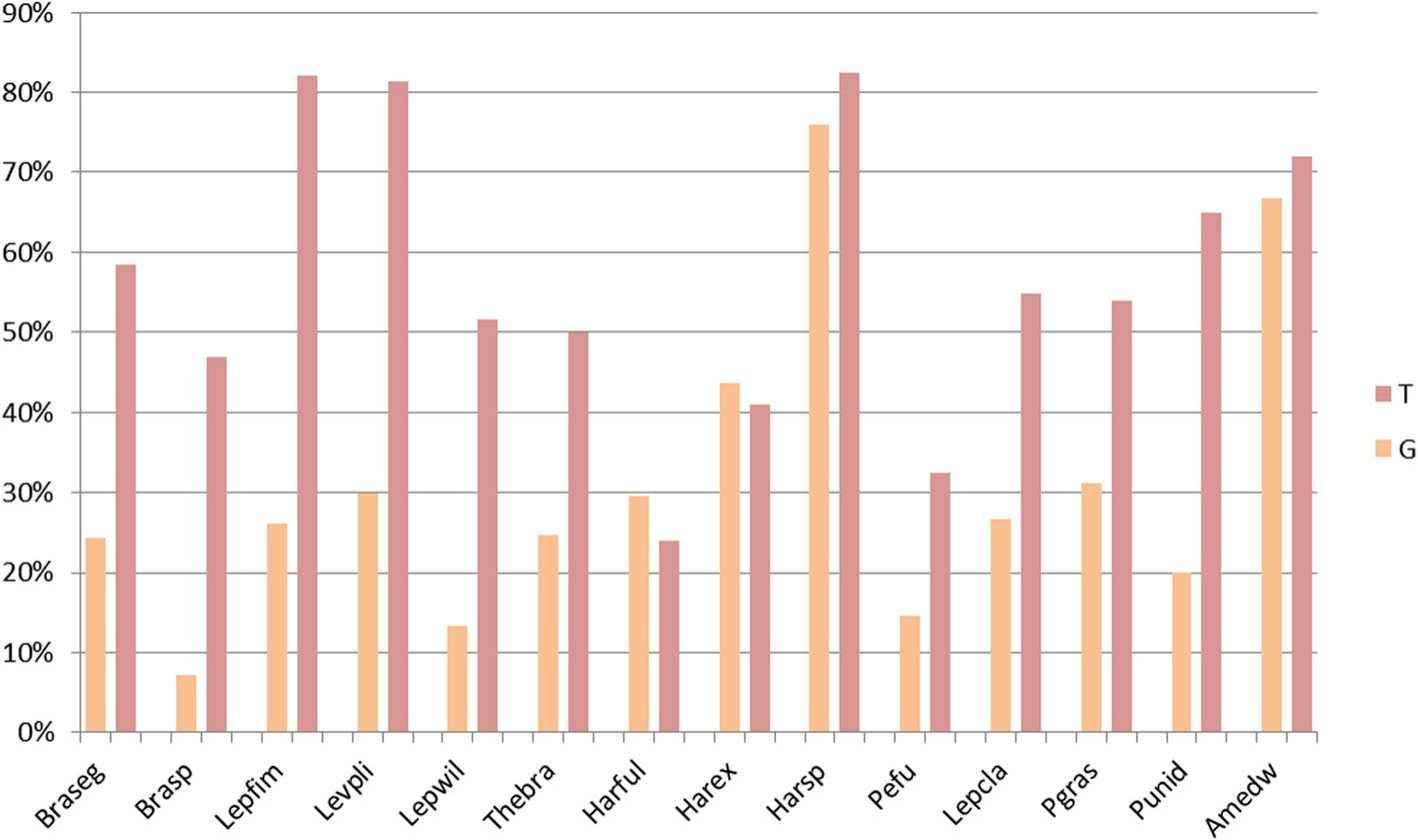
Number of LTR–retrotransposons only detected in low-coverage genome or in transcriptome. For each species, the proportions of unshared genomic TE sequences only observed in the genomes (G) are indicated in orange, and the proportions of unshared genomic TE sequences only observed in the transcriptomes (T) are indicated in red. Species are ordered according to their phylogenetic relationships as in Fig. 4.

**Fig. 6:**
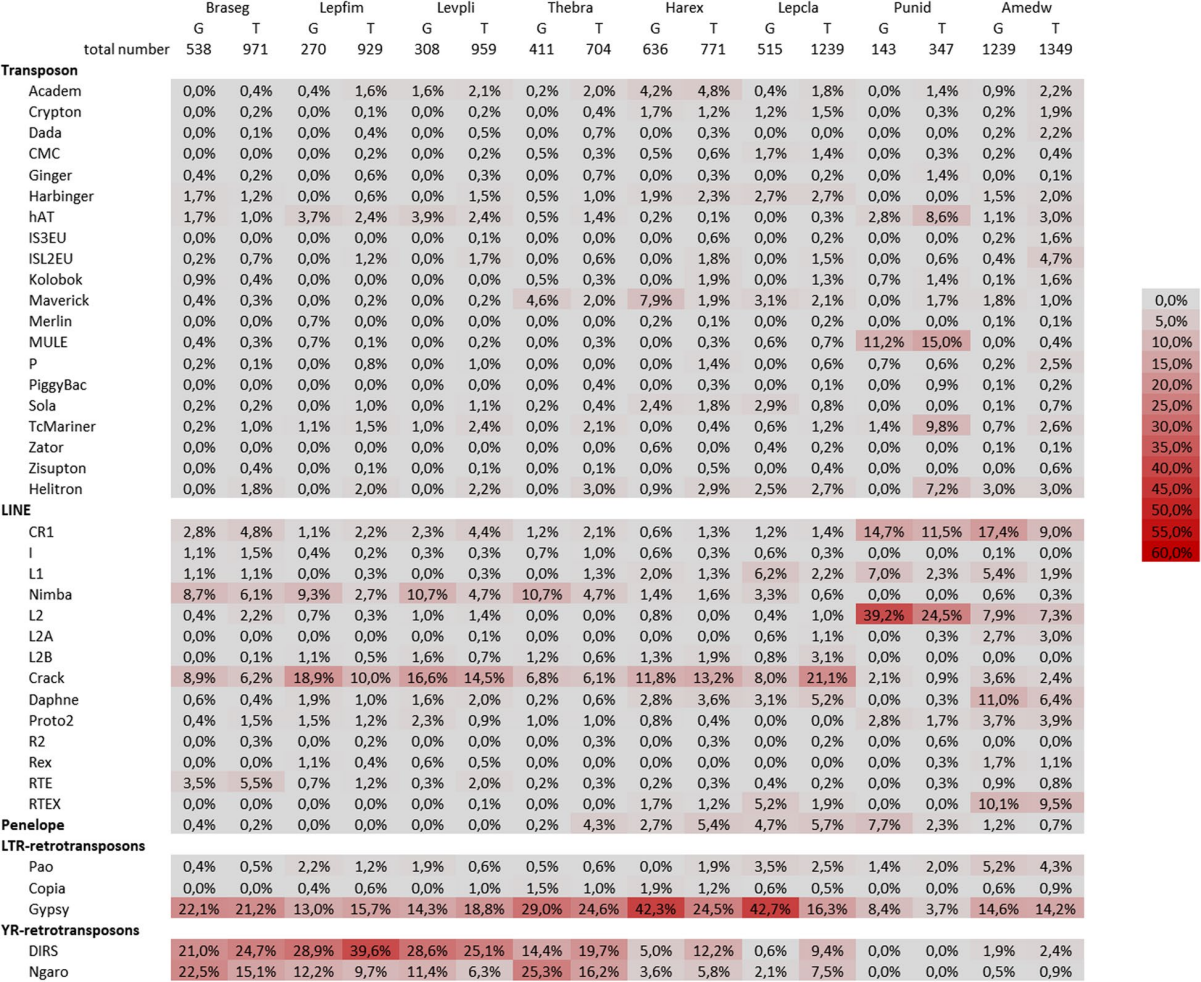
Heat map of TE types identified in the low-coverage genome or in the transcriptome. For each of the eight representative annelid species, element proportions in the genome (G, left column) or transcriptomes assembled from 40 million reads (T, right column) are shown as in Fig. 3. The total number of TE families detected is indicated above each column. The columns Species are ordered according to their phylogenetic relationships as in Fig. 4*.* Braseg – *Branchinotogluma segonzaci*; Lepfim - *Lepidonotopodium fimbriatum*; Levpli - *Levensteiniella plicata*; Thebra - *Thermopolynoe branchiata*, Harex - *Harmothoe extenuata*; Lepcla - *Lepidonotus clava*; Punid - *P. unidentata*; Amedw - *Neoamphitrite edwardsii*

Focusing on LTR-retrotransposons, we searched for sequences only found in low-coverage genomes or in transcriptomes (Fig. 5). For all the species the percentage of TE transcripts not recovered in the genomic consensus (in red) is larger or quite equivalent to the proportion of genomic consensus not found in the transcriptome (in orange). In most species less than one third of the genomic sequences were not found in the transcriptome with the exception of *Harmothoe extenuata* (44%), *A. edwardsii* (67%) and *Harmothoe* sp*.* (75%). Conversely, more than 50% of the TE transcripts were not found in the low-coverage genome with the exception of the polynoids *Branchinotogluma* sp. (47%), *H. extenuata* (41%), *Pettitbonesia furcosetosa* (32%) and *H. fuligineum* (24%). To compare the relative proportions for the different types of elements obtained for the two datasets (Fig. 6 and Additional file 6), we have considered weak (> 5%), moderate (ranging from 10 to 15%) and high variations (> 15%). For 9 annelids only limited differences are observed, restricted to weak variations that concern only one to three types of elements per species. *Lepidonotopodium fimbriatum* (Lepfim) shows slightly more differences with 10.7% more transcriptomic DIRS sequences*. P. unidentata* (Punid) also appears a bit unusual because it exhibits a strong excess of genomic L2 sequences (difference of 15%). For the remaining four species (*H. extenuata* (Harex), *H. fuligineum* (Harful), *P. furcosetosa* (Pefu) and *L. clava* (Lepcla)), we observed high variations between the two methods linked to an excess of Gypsy in the low-coverage genomic data, and moderate excess of Crack and, to a lesser extent, of DIRS in the transcriptomes. These marked discrepancies are therefore limited to 4 phylogenetically-related species that exhibit similar patterns of differences. An increase in the number of Gypsy, although more moderate (8%), is also observed in *Harmothoe* sp.. They are moreover very probably related to a fractioning of TE consensus in the genomic data. Indeed, these species have an excess of genomic sequences when we compare the number of sequences found in both the genome and the transcriptome (e.g. 329 consensus genomic sequences vs 180 transcripts in common in *H. fuligineum* and 179 consensus genomic sequences vs 118 transcripts in common in *L. clava*), although these values should be similar. Together, these results show that the transcriptomic data reflect well the TEs diversity observed at the low-coverage genomic level.

### Phylogenetic relationships among LTR retrotransposons in polychaetous annelids

As LTR retrotransposons represent the dominant fraction of the TEs identified in most annelid genomes, we have taken a closer look at their diversity and evolution. Transcriptome analyses of 26 annelids revealed a high number of potential families with 199 Copia, 491 BEL/Pao and 5437 Gypsy (Table 1). The most striking fact is the great heterogeneity observed according to the host groups. While Terebellidae and Polynoidae have a high number of families (about 250 on average), LTR-retrotransposons are less diversified in Alvinellidae with a maximum of 69 families in *P. grasslei* and less than 20 families in 4 of the 5 other species. In particular, two species, *Alvinella caudata and Paralvinella fijiensis,* have very few LTR-retrotransposons with only four Gypsy elements and one BEL/Pao element, respectively. Conversely, the only representative of Ampharetidae, *Melinna palmata*, which appears to be the closest group to Alvinellidae, exhibits a very high diversity with nearly 800 families, including more than 600 Gypsy.

**Table 1:** Number of families of LTR retrotransposons detected in transcriptomes of polychaetous annelids

Polynoidae	index	Copia	BEL/Pao	Gypsy	
*Branchinotogluma segonzaci*	Braseg	0	5	206	**211**
*Branchinotogluma trifurcus*	Bratri	11	0	256	**267**
*Branchinotogluma* sp*.*	Brasp	13	0	392	**405**
*Lepidonotopodium fimbriatum*	Lepfim	6	11	146	**163**
*Levensteiniella plicata*	Levpli	10	6	180	**196**
*Lepidonotopodium williamsae*	Lepwil	11	0	344	**355**
*Thermopolynoe branchiata*	Thebra	7	4	173	**184**
Eulagiscinae gen. sp*.*	Eula	7	2	102	**111**
*Alentia gelatinosa*	Alge	1	2	59	**62**
*Harmothoe crosetensis*	Harcros	17	0	180	**197**
*Harmothoe fuligineum*	Harful	16	0	218	**234**
*Harmothoe extenuata*	Harex	9	15	189	**213**
*Harmothoe* sp*.*	Harsp	13	13	218	**244**
*Pettitbonesia furcosetosa*	Pefu	15	10	354	**379**
*Lepidonotus clava*	Lepcla	6	31	202	**239**
**Alvinellidae**					
*Alvinella caudata*	Acaud	0	0	4	**4**
*Paralvinella grasslei*	Ppalm	0	15	54	**69**
*Paralvinella palmiformis*	Pgras	1	11	53	**65**
*Paralvinella fijiensis*	Pfiji	0	1	0	**1**
*Paralvinella hessleri*	Phess	1	0	13	**14**
*Paralvinella unidentata*	Punid	0	7	13	**20**
**Ampharetidae**					
*Melinna palmata*	Mepal	20	160	619	**799**
**Terebelidae**					
*Amphitrite edwardsii*	Amedw	12	58	191	**261**
*Amphitritides* sp*.*	Amphi	7	21	89	**117**
*Terebella lapidaria*	Terlap	9	99	349	**457**
*Thelepus* sp*.*	Thelep	7	19	141	**167**
		**199**	**490**	**4745**	

As expected, the proportions of the 3 superfamilies are very close to the proportions observed in mollusk genomes: 87% Gypsy, 9% BEL/Pao and 4% Copia [27]. On the other hand, here again, strong differences can be underlined between host groups. In particular, Polynoidae only have a small number of BEL/Pao elements (≈ 3% on average), whereas Copia elements are almost absent from Alvinellidae (only 2 families described), as well as for the Polynoidae *B. segonzaci* and *Alentia gelatinosa*.

**Fig. 7:**
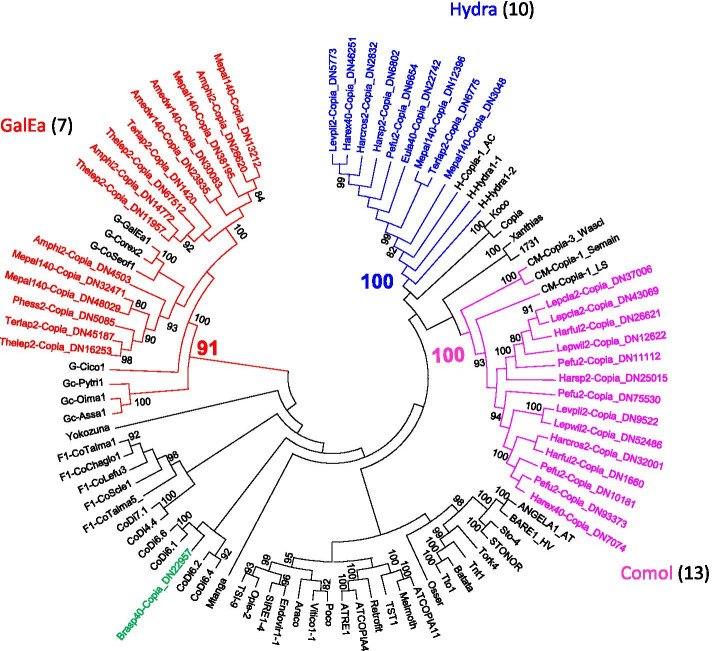
Phylogenetic relationships of Copia retrotransposons. The tree is based on Neighbor-Joining analysis of RT/RNaseH domain amino acid sequences. The Copia families from annelids are indicated in color, as are the major clades to which they belong. The number of annelid species covered by each clade (evaluated on both RT/RNaseH and Integrase domains) is given between brackets. Node statistical support values (> 70%) come from non-parametric bootstrapping using 100 replicates

**Fig. 8:**
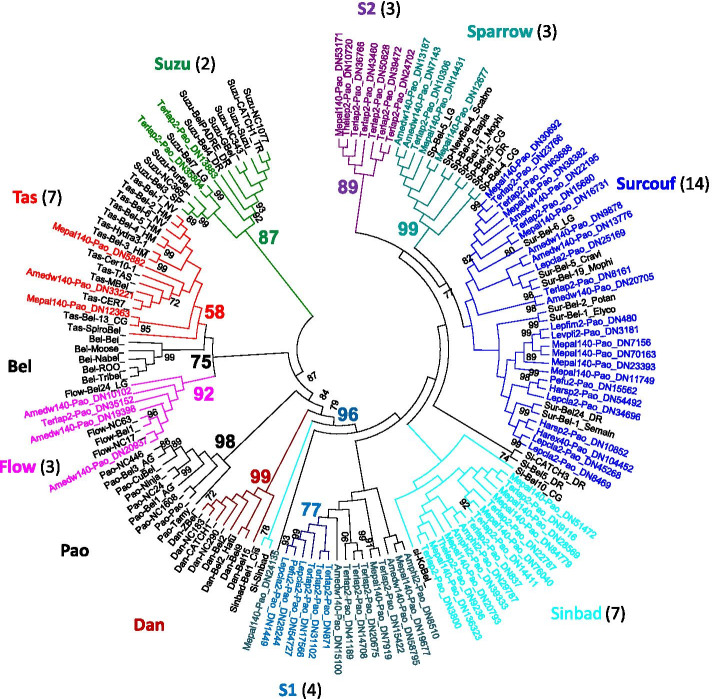
Phylogenetic relationships of BEL/Pao retrotransposons. The tree is based on Neighbor-Joining analysis of RT/RNaseH domain amino acid sequences. The BEL/Pao families from annelids are indicated in color, as are the major clades to which they belong. The number of annelid species covered by each clade (evaluated on both RT/RNaseH and Integrase domains) is given between brackets. Node statistical support values (> 70%) come from non-parametric bootstrapping using 100 replicates

**Fig. 9:**
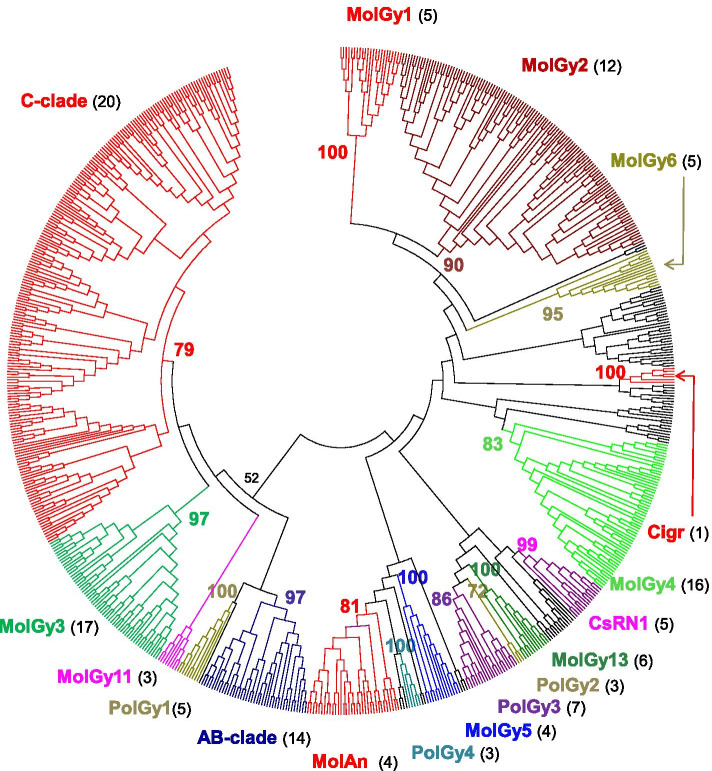
Phylogenetic relationships of Gypsy retrotransposons. The tree is based on Neighbor-Joining analysis of RT/RNaseH domain amino acid sequences. The Gypsy families from annelids are indicated in color, as are the major clades to which they belong. The number of annelid species covered by each clade in the phylogeny is given between brackets. Node statistical support values (> 70%) come from non-parametric bootstrapping using 100 replicates

To apprehend the diversity of annelid elements in terms of clades, several phylogenetic trees have been built (Figs. 7 to 9). For this purpose, the sequences of LTR-retrotransposons coming from transcript assemblies were translated for the RT/RNaseH domain. As we only retained sequences covering at least 80% of this domain, a very large number of incomplete elements or elements with sequences corrupted by frameshifts were discarded from the analysis. To have a thorough view of the phylogeny of the Copia and BEL/Pao elements present in restricted copies, additional trees were built based on the Integrase domain (Additional files 7 and 9). Thus for Copia and Bel/Pao elements the total number of families identified is estimated on the whole of the two phylogenies (only considering the transcripts found on both the RT/RNaseH and Integrase domain once). In total, 94 Copia (in addition to 41 reference elements), 177 BEL/Pao (in addition to 63 reference elements) and 550 Gypsy (in addition to 167 reference elements) were used. Clades were defined on the same two criteria used in our earlier analysis in mollusk genomes [27]: (i) to be shared by several species and (ii) to form a monophyletic group with a bootstrap value greater than 70%.

For Copia elements, the results obtained from the RT/RNaseH or Integrase domains are very similar (Fig. 7 and Additional file 7). Considering potential contamination, it seems important to validate the phylogenetic relationships of each element with elements already described in the literature. For example, we have considered with caution the rare Copia elements attached to clades previously defined from plants elements or other taxa. With the exception of four isolated sequences, all the Copia elements of annelids are almost equally distributed in the three clades already known: GalEa (23 families), Hydra (23 families) and Comol (34 families). The other clades of Copia elements described in arthropods, and more specifically in insects (Copia, Tricopia, Mtanga, 1731, Humnum, as inferred in the GypsyDatabase) were not found here, as it was the case with the mollusks. The most striking result remains the fact that the CoMol clade, previously defined from only 4 mollusk elements, is the most represented in annelids, detected in half of the species. It could therefore also be present in other taxa that have yet to be identified, and thus constitute one of the major clades of Copia in metazoans. The GalEa clade, known to be the most widely distributed within metazoans, has also been found in most annelids. Finally, the presence of Hydra elements in several annelids supports the hypothesis that this clade may have a wide distribution among metazoans.

Considering the BEL/Pao elements, both trees on each domain also give roughly the same results (Fig. 8 and Additional file 8). No elements of the eponymous BEL and Pao clades are detected in annelids, as well as for the Dan clade. However, the other six known BEL/Pao clades can be identified at different levels of importance. The Flow clade, presumed to be relatively rare in metazoans, is clearly present in a few annelid species (6 families in 3 species). Considering the clades usually more widely distributed in metazoans, in annelids the Suzu clade is poorly represented (2 of our species) whereas the Tas elements appear as the second most important clade (7 species).. The major clarifications provided by the annelid LTR-retrotransposons regard the Sailor lineage. This well supported group (bootstrap value of 96) dominates the annelids BEL/Pao families. It is composed of 5 clades: i) two new small clades which are poorly represented and only comprising annelid sequences, S1 (bootstrap value greater than 77, 8 families, 4 species) and S2 (bootstrap value greater than 89, 8 families, 3 species); ii) the Sparrow clade, which is strongly supported but not very frequent in annelids; iii) the Surcouf clade is the clade that clearly dominates (27 families, 14 species), it remains supported when considering the Integrase domain (bootstrap value of 70, Additional file 8), even if the bootstrap value decreases with the RT/RNaseH domain (bootstrap value of only 57); iv) lastly, the Sinbad clade is only supported by the Integrase domain (bootstrap value of 87), but this grouping is not monophyletic using the RT/RNaseH. There are also in this Sailor group about ten isolated sequences, which cannot be attached to any clade.

The tree obtained for the Gypsy superfamily reveals 17 clades in annelids (Fig. 9). A simplified representation of the Gypsy diversity is available and shows annelid elements of the same clade as compressed subtrees (Additional file 9). This better differentiates the reference Gypsy elements and therefore allows to determine whether a clade has been previously reported or not [27]. Among the clades referenced in the GypsyDatabase only five are well recovered (clades A and B (again indistinguishable from each other), C-clade, Cigr and CsRN1). Concerning the MolGy clades previously defined solely from mollusk elements, 14 of the 16 clades are also present in annelids; while the MolGy7 and MolGy10 are missing. The most striking point is that the 8, 9, 12, 14, 15 and 16 small MolGy clades (with unclear affinities) previously reported form a well-supported single clade we called MolAn (**Mollusk**s and **An**nelids, bootstrap value of 81, Additional file 9). Finally, four new putative clades can be further identified with annelid elements (PolGy 1 to 4). If we look more closely at the number of families, the C-Clade largely dominates with a quarter of elements that occur in at least 20 species. Thus, within the Mag lineage, elements of the large clades A and B, MolGy3, MolGy11 and of the new small clade PolGy1 are also well distributed among the annelids. The clades MolGy2 and MolGy 4, detected in 12 and 16 species, respectively, also appear quite abundant while all the other clades appear in at most 7 species.

### Distribution of LTR-retrotransposon clades in annelid species

**Fig. 10:**
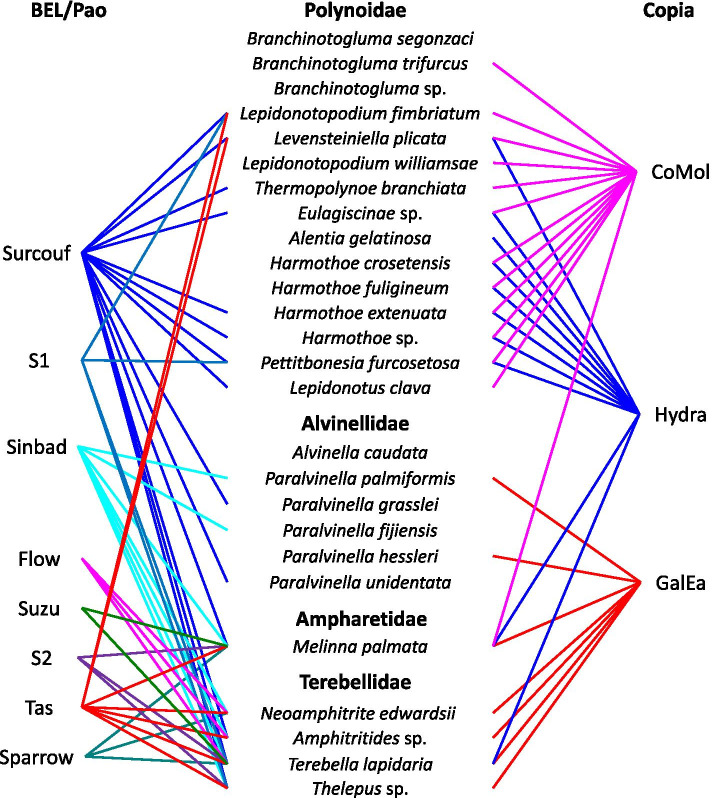
Distribution of Copia and BEL/Pao clades within annelids. Tanglegram-like representations of annelids and element clades with an ordered list of species names according to their phylogenetic relationships and LTR-retrotransposons clades plotted on the sides. A connection is drawn when a TE clade is found in a given species and each connecting lines are coloured according to clade description

As phylogenetic trees revealed major and minor clades containing a variable number of elements, we checked whether this feature could also be true in terms of distribution among host species. The host species associated with each clade are shown in Fig. 10. As only transcripts with large translated RT/RnaseH domain were included in the phylogenetic trees, an absence of detection does not mean that a clade is not present in a given host. It seems that the phylogeny of the hosts has a great influence on the distribution of the different clades, especially for Copia and BEL/Pao elements (Fig. 10). The presence of Copia elements is confirmed in 20 annelid species. The three clades appear to have different patterns of distribution. The GalEa clade is presently not represented in Polynoidae but is found in other species that possess Copia elements. Conversely, the Hydra and CoMol clades are found in most Polynoidae, but are otherwise detected in only one Terebellidae and in the ampharetid worm. This latter is the only species that has the three Copia clades. The presence of BEL/Pao elements is confirmed in 17 annelid species (Fig. 10). It seems that their diversity appears mainly within the Terebellidae and Ampharetidae, in which all 8 clades are represented. On the contrary, in Polynoidae and Alvinellidae only the Surcouf clade is widely distributed and only three other clades appear sporadically: Sinbad in two Alvinellidae, Tas and S1 in two Polynoidae. Gypsy are by far the most widespread LTR-retrotransposons in annelids. Among the 17 clades, 9 do not display specificity with respect to host phylogeny (Additional file 10). The three clades Molgy1, MolGy5 and MolAn are presently restricted to the Ampharetidae and Terebellidae. Four other clades have only been confirmed in Polynoidae: the CsRN1 clade; and three of the new annelid clades, PolGy1–3. In conclusion, Alvinellidae clearly displayed the lowest diversity of LTR-retrotransposons with only 8 identified clades (1 Copia, 2 BEL/Pao and 5 Gypsy). Polynoidae have a higher diversity with 18 clades recognized (2 Copia, 3 BEL/Pao and 13 Gypsy). On the other hand, the ampharetid worm *M. palmata* presents a very high diversity with at least 19 clades (3 Copia, 6 BEL/Pao and 10 Gypsy) as for some Terebellidae such as *Terebella lapidaria* which displays 22 of the 28 clades of the LTR-retrotransposons.

### Coexpression of TE and their regulators

**Fig. 11:**
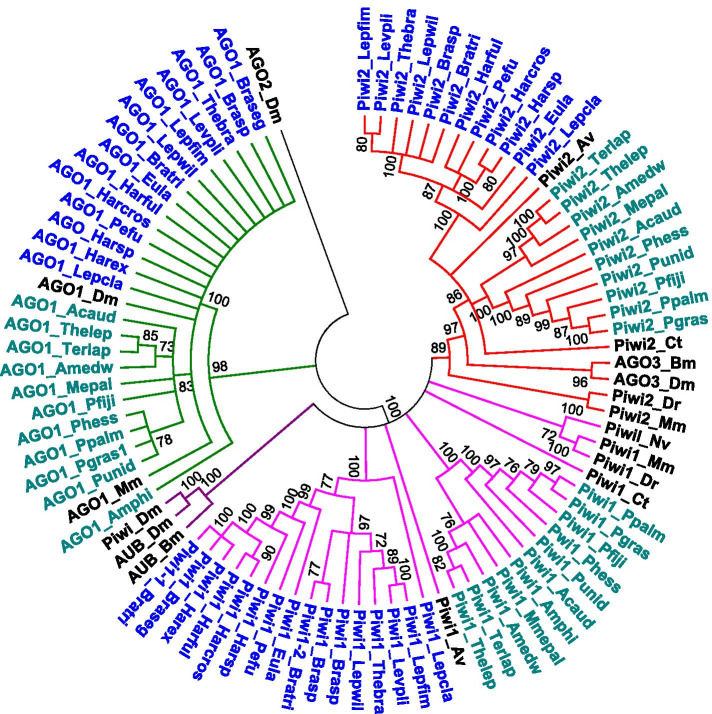
Phylogenetic relationships of Argonaute proteins. The tree is based on Neighbor-Joining analysis of amino acid sequences. Annelid proteins identified in this study are colored: blue for the Phyllodocida, blue-green for the Terebellida. The major clades are also presented in different colors. Node statistical support values (> 70%) come from non-parametric bootstrapping using 100 replicates. Species abbreviations: Av, *Alitta virens*; Bm, *Bombyx mori*; Ct, *Capitella teleta*; Dm, *Drosophila melanogaster*; Dr., *Danio rerio*; Mm, *Mus musculus*; Nv, *Nematostella vectensis*

The identification of transcriptomes containing numerous TE sequences in polychaetes, irrespective of their environments, raised the question of how these genomes are facing such potential threat and led us to focus on TEs control machinery. In particular, looking at the diversity of TEs across the transcriptomes of various annelids, we wondered if they all shared a similar status with respect to possible regulation by PIWI interacting RNAs (piRNAs). Thus, we took advantage of the set of transcriptomes to investigate the diversity of PIWI proteins in Phyllodocida and Terebellida (Fig. 11). The PIWI family of Argonaute proteins and their associated small RNAs have been shown to be involved in the repression of TEs transposition [43]. They are synthesized by loci enriched in fragments of TEs called piRNA clusters. PIWI/piRNA complexes target TE transcripts by base pair complementarity. piRNA biology has been extensively studied in gonads of model organisms such as mice [44] and *Drosophila* [43], and have been identified in a variety of arthropods [45] and in sea anemones [46], in which they were also detected in somatic tissues. In parallel, PIWI proteins were found to be expressed in several taxa, including vertebrates, arthropods, nematodes and annelids [47–50], but data on their expression in polychaetes was scarce.

Our results indicate that all annelid species harbor PIWI proteins, often as two distinct copies, Piwi1 and Piwi2, inferred to be an ancestral gene duplication, as for the polychaetes *C. teleta* [49] and *Alitta virens* [47] (Fig. 11). However, Piwi2 seems to be absent in *A. gelatinosa*, *B. segonzaci* and *H. extenuata* and Piwi1 was found duplicated in *Branchinotogluma* sp*.* and *B. trifurcus*. Piwi2 proteins form a well-supported subgroup (bootstrap value of 89), which includes representatives from a broad range of metazoan taxa, while there is some lack of resolution among the Piwi1 proteins, as previously reported [49]. A notable result is that whatever the protein considered, we observe a very strong influence of the phylogeny of the host species with a clear separation into two supported clades of Phyllodocida proteins on the one hand, and of Terebellida proteins on the other hand. Interestingly, during our analysis, we came across 18 large transcripts (> 8 kb) highly enriched in fragments of various TEs evocative of piRNA cluster structures (Additional file 11). To conclude, our data suggest that in polychaetes, PIWI proteins and putative piRNA clusters are concomitantly expressed with a large variety of TEs allowing a constant adaptive immunity against TEs.

## Discussion

### Estimation of TEs diversity using transcriptomes

Although the combination of genomic and transcriptomic data constitutes an efficient approach to study transposable elements, we show that transcriptomic assemblies are sufficient to access the number of families and their diversity as shown by our results on LTR-retrotransposons. The use of transcriptomes alone is cheaper, especially for species with very large genomes, and can be used to study a larger number of species simultaneously. Such a point is very important as the number of sampled species does matter a lot to get the most appropriate picture of the TEs distribution among species in comparative purposes between very distinct phylogenetic lineages. In addition, transcriptome analysis gives access to the fraction of expressed TEs that are still potentially mobile and therefore involved in the evolution of genomes. However, it should be noted that, especially for LTR-retrotransposons, not all autonomous copies are necessarily found in the transcriptome under basal conditions [51, 52]. Conversely, the transcriptional expression of an element is not necessarily followed by transposition events [7]. Moreover, even if we did not consider TE fragments of very small size (< 150 bp), deleted copies can also be found in the transcriptome. The representativeness of the data obtained from transcriptomes alone can be assessed by comparison with those obtained on genomes. Both the deep analyses of the LTR retrotransposon sequences and the diversity of different types of elements provide similar results. In fact, the only notable difference was found in species of the *Harmothoe* group, in particular regarding the Gypsy elements. However, this observation may result from an artifact. Indeed these species display some important differences between the numbers of shared LTR-retrotransposons in the genomic and transcriptomic compartments, with a global overestimation of the number of elements identified in genomic data, possibly due to a fragmentation of these elements. Nevertheless, our results clearly indicate that a large proportion of the TE diversity found in annelid genomes is expressed. Of course a perfect match is impossible since (i) in both approaches the sequencing depth does not allow us to obtain complete sequences and some elements can be split into two or more fragments, and conversely some of them may be lost during assembly, (ii) rare elements with highly expressed copies will only be found in the transcriptome; reciprocally, the inventory of elements expressed at low level in transcriptome may not be exhaustive as they may not all have a sufficient level of expression for detection and/or assembly.

Keeping this in mind, we observe a very large quantity of expressed elements, and some of the sequences obtained are very large and probably correspond to almost complete transcripts. Interestingly, more TE sequences are detected in the transcriptomes, and many were missing from the low-coverage genomes. This phenomenon has already been observed, for example in the mosquito *Anopheles funestus* [40]. In this latter study, out of the 211 elements characterized in the mosquito transcriptome, 30% were not recovered in the genome. We obtain a comparable ratio in our study using low-coverage genomes whereas they used an assembled and presumably complete one [53]. The lack of genomic equivalent of a high percentage of TE sequences identified in transcriptomes does not only reflect a discrepancy due to low-coverage sequencing since it can also be observed, to a lesser extent, with complete assembled genomes [40]. Reciprocally, these latter authors only reported that 40% of genomic elements were found in the mosquito transcriptome, compared to 70% for annelids. But again, the use of a complete assembled genome should allow to detect elements present in small copy numbers that may be expressed at low level; a category of elements that may be missed through low-coverage genome analysis. In both cases, transcriptomes can give access to elements not detected by the genomic approach but whose expression is likely to be high. And more importantly, it allows a significant increase in the number of species that can be studied because many transcriptomes, with greater or lesser sequencing depths, are available for many animals in a wide range of non-model taxa.

### Influence of the number of reads during assembly

To our knowledge, this is the first comparative description of various types of TEs based solely on several transcriptomes. Even if various other annelid transcriptomes are freely available (for data resource example see https://bitbucket.org/wrf/polychaete-transcriptomes), we chose to focus our study only on two groups of polychaetes while optimizing the number of species in each of them. Furthermore we wanted all transcriptomes to be produced under exactly the same conditions with as much coverage as possible for our comparative analysis. Our different results clearly show that this approach is applicable to studies of organisms with limited genomic resources and allows the description of a large number of elements for all the types and families studied. Finally, in the specific case of the detailed study of particular elements such as LTR-retrotransposons, the data and conclusions obtained are as informative and important as those obtained with the use of assembled genomes. And the clear influence of host phylogeny, at the level of annelid orders, supports the validity of these observations.

One of the major issues that arose during this type of analysis is the quality of the de novo assemblies, and consequently the estimation of an optimal number of reads to obtain reliable data. This question is reminiscent with study using low coverage genome, in which the authors often optimized the length of TE sequences on the basis of the N50 [37]. It is also relevant for transcriptome analyses of genes and their variants [41, 42] or differential expression analyses [54]. It has been suggested that sequencing of very small numbers of reads can be most subject to biases [55]; and previous studies have shown that representative assemblies are difficult to generate below 20 million reads [42]. In fact, there is a trade-off between the quality of information and the time and financial costs. For instance, few reads may give less accurate results, with some elements possibly divided into several sequences, or even some elements not detected. Conversely, some assemblers such as Trinity can become ‘verbose’ if the number of reads is increased too much and result in the reconstruction of chimeric sequences. This type of discrepancy can be partially avoided by filtering the transcripts obtained based on the proportion of reads that map to a sequence. We deliberately chose to not apply this type of filter in this study because we wanted to keep as much information as possible without adding extra steps, keeping in mind that we could have false positives. Moreover, in the study of LTR-retrotransposons such artifactual sequences will be filtered by the phylogenies which are based on full length translated RT/RNaseH or Integrase domains and the rare intra-domain chimeras would be revealed by the phylogeny.

We therefore compared the data obtained on TEs identified on 15 distinct annelid transcriptomes with an increasing sequencing effort:

(i) The proportions of the different TE superfamilies show no clear influence of the number of reads. The only noteworthy point is the slightly more marked differences between the 20 million-read assemblage and the others.

(ii) The superfamilies poorly represented in some species (between 0 and 3 elements depending on the assembly considered) were also checked (BLASTX on Repbase). Of a total of 258 sequences only 16 did not correspond to the predicted superfamily. These sequences were mostly found in transcriptomes obtained with 100 million or more reads. On the other hand, with only 20 million reads these rare superfamilies were no longer detected in one third of the cases (species/TE superfamilies association).

(iii) The third analysis deals with the increase of the number of elements, and thus the nature of the new sequences detected when the number of reads increases. To address this issue, the 152 Copia sequences obtained with 40 million reads (assembly-40) were compared with the 217 sequences obtained with the maximum number of reads (assembly-Max, i.e. 140 million for 13 species and 100 or 120 million for six others). Only 103 sequences are common between these two datasets, of which ten are separated into two fragments in assembly-40. Curiously, in both cases we found nearly 20% of specific sequences (25 and 41 sequences respectively), as well as several other unshared sequences (6 and 24, respectively) but too short (< 400 bp) to know whether or not they are isolated fragments of an element already described. However, from the assembly-Max we also observe 6 sequences that have no link with Copia elements, as well as 34 redundant sequences that appear in a second cluster although they clearly belong to a family already counted (the latter corresponds to sequences with a large insertion or merged with an unknown sequence, perhaps as the result of an assembly error). So in balance, the use of assembly-Max allows the detection of only 16 additional exploitable Copia elements when compared with the assembly-40, but also adds 40 artifactual elements.

In conclusion, it is clear from our combined results that a number of about 40 million reads seems optimal for this type of TEs analysis because it allows a good balance between the quality of the data, the noise and the sequencing effort. Even if this criterion of 40 million has only been established on assemblies of annelids, it seems to be valid for other organisms as suggested by two other studies. This number is in agreement with those suggested to study gene expression. Indeed, a comparative transcriptomic study across 10 invertebrates was used to generate a tractable catalog of annotated genes [41]. Through a saturation analysis the authors concluded that the sequencing efforts (16.4 to 51.7 million with a mean of 39.5) were sufficient to accurately estimate the completeness of their transcriptome datasets. A second study looked for an optimal sequencing depth for de novo transcriptome assembly in order to gather information about genes and their expression [42]. Using mainly marine organisms, including the polynoid *Harmothoe imbricata*, they concluded that representative assemblies may be generated with as few as 20 million reads or 30 million reads for RNA-level coverage. On the other hand, it cannot be taken for granted that the largest set of reads will produce the best contigs; and using conserved genes as a metric, there appears to be limited benefit of sequencing beyond 60 million reads as the discovery of new genes is low and sequencing errors of highly-expressed genes are likely to accumulate.

### Cladistic analysis of annelid LTR-retrotransposons

Several recent studies have highlighted the differences in abundance and diversity of LTR-retrotransposon superfamilies within fungi and metazoans [21, 24, 29]. The study of LTR-retrotransposons in mollusks allowed us to characterize several new clades and confirms strong inequalities in the diversity within Copia, BEL/Pao or Gypsy superfamilies [27]. This latter study raised several major questions: what is the real evolutionary success of these different clades and how are they distributed within metazoans? Is the number of clades limited or can it increase with each new phylum study, especially for Gypsy elements? Studies of another phylum, such as annelids, may allow us to provide some answers to these questions.

The number of TE families detected in annelids (Table 1) is greater than in mollusks; by a factor of 1.5 for Copia and BEL/Pao, and by a factor of 4 for Gypsy. Several factors may interact to explain such difference; i) there are indeed more TE families in the annelids, because annelids have for example larger genomes [56], ii) the number of TE families may be slightly overestimated if the transcript of an element is broken into several fragments, and iii) the number of studied species is different. Here we analyzed the entire set of transcribed sequences larger than 150 bp in 26 annelid transcriptomes compared to our previous study on only 9 molluscan genomes in which only the complete, and therefore probably recent, copies of TEs have been taken into account. Elements from 46 additional species of mollusks were however obtained from the analysis of the databases, but for these we had no information on the nature of the data made available (number of transcripts deposited in the various databases), and again only families identifiable by an intact RT/RNaseH were taken into account. These results highlight how difficult it is to make comparisons on a quantitative basis between two different studies, as the definition and outcome of “families of elements” can vary considerably from one study to another. Regarding phylogenetic trees, a large number of annelid transcripts were not exploitable with the RT/RNaseH domain alone. This type of difficulty had less impact in the study of mollusk elements because, starting from complete genomes, we had more integral sequences for each family for which this specific domain was often present. We therefore completed our annelid study with trees based on the Integrase domain. In the end, the phylogenetic analyses yielded similar numbers of elements (94 Copia elements in annelids vs 93 in mollusks, 177 BEL/Pao elements vs 248, and 550 Gypsy elements vs 989).

A previous analysis of the *L. luymesi* genome [36] identified two Copia elements, one of which belonging to the GalEa clade, two Bel/Pao elements belonging to the Sinbad clade, and several Gypsy elements from the CsRN1 clade, the AB-clade and the C-clade, One Gmr1 element was also detected, belongings to a clade not found in our analysis, They also described five new clades: the LGF7, LGF8 and LGF9 clades, which are part of the Mag lineage, that could correspond to the PolGy1, MolGy3 or MolGy11 clades that we identified, and the LGF2 and LGF4 clades that could correspond to other MolGy and PolGy clades. The LTR-retrotransposons of new annelids provide important additional information for the Copia, BEL/Pao and Gypsy superfamilies. In the case of the Copia elements, it was hypothesized that the CoMol clade may have recently emerged in mollusks. Our study indicated that this clade is older and widespread, and that the GalEa and/or Hydra clades are not dominant in all metazoan phyla (excluding insects) as observed in crustaceans [24] and mollusks [27]. Given the presence of many families of the Hydra clade in both mollusks and annelids, it remains to be determined how these elements may be distributed in other phyla. In the case of BEL/Pao elements, the results are quite comparable to those obtained in mollusks. The BEL and Pao clades still remain restricted to insects; and while some Dan elements had been identified in mollusks, they are lacking in the transcriptomes of annelids. Conversely, the small Flow clade seems to have a fairly wide distribution within metazoans (cnidarians, planar, mollusks and annelids). Even if the Sailor lineage still largely dominates in both mollusks and annelids, the main clades are different between mollusks (Sparrow) and annelids (Surcouf). While it was difficult to extrapolate the existence of these two new clades outside mollusks, their presence in annelids confirms that they are not limited to a single phylum. On the contrary, the characterization of the Sinbad clade appears more complex. Either we can consider that this Sinbad clade is indeed maintained but that the dataset here is not sufficient to support it. Or it is possible that this clade, already poorly supported in mollusks, is artifactual and sometimes gathers various isolated elements (11 Sailor elements of annelids could not be attached to any clade). In the latter case, the historic name Sinbad could be used for the entire lineage instead of Sailor. When studying mollusks, only 6 of the Gypsy clades referenced in the GypsyDatabase [31] were found and no less than 16 new potential clades MolGy were characterized. We then suggested that only a limited number of major clades, including perhaps some of the most important new clades such as MolGy1 and/or MolGy2, could be more widely distributed within metazoans. Conversely, the large number of both families and clades suggested that extending the study to other host taxa would lead to the characterization of many new phylum-specific clades. These two hypotheses are clearly challenged by our current results. Only the Tor2 clade (poorly represented in mollusks) was not detected in annelids, almost all MolGy clades were thus recovered in annelids, and finally only 4 new phylum-specific clades were observed. These three arguments strongly suggest that, even if the number of Gypsy clades remains much higher than that of Copia or BEL/Pao in metazoans, the number of elements already captured is probably reflecting well the true diversity of this group. Additionally, this assumption implied that the number of phylum-specific clades may be in fact limited (for the moment, 2 in mollusks and 4 in annelids). In more detail, the C clade, and more generally the clades of the Mag group, still seems to be the most common Gypsy clade of metazoans. The MolGy clades do not necessarily have the same importance, in terms of number of families and distribution among the hosts, between annelids and mollusks; for example, the clades MolGy1 and MolGy6 are very little represented in annelids. Regarding the grouping of six MolGy clades within the same new MolAn clade, even if these clades appeared to be phylogenetically related in mollusks there was then no argument for grouping them together. Only the addition of annelid elements makes possible their clustering into a well-supported monophyletic new group of Gypsy.

It is important to note that in order to better apprehend the revision of our various hypotheses and set up new conclusions on the diversity and distribution of LTR-retrotransposons within metazoan, it is necessary to take into account the fact that mollusks and annelids are two fairly closely-related phyla branches within Spiralia. New information from other phyla (e.g. Cnidaria and /or Echinodermata) is still needed to provide a clearer definition of the number and distribution of clades for each of the three TE superfamilies.

## Conclusion

With this study we have shown that the use of transcriptomes assembled from 40 million reads was sufficient to have access to a very large part of the transposable elements compared to those obtained by low coverage sequencing. This allowed us to carry out the first comparative analysis of TEs in annelids, focusing on the LTR-retrotransposons which appear to be the most abundant Order in most of the genomes. We characterized different clades defined by 1021 LTR-retrotransposon families identified in 26 polychaetous annelids. The clades observed are similar to results previously obtained on mollusks. The Gypsy elements were unequivocally dominant but we have identified only 17 clades of which only 4 are new, suggesting that the number of Gypsy clades, although high, may be more limited than we previously thought in metazoans. The BEL/Pao elements were clearly the second-most abundant superfamily, especially because of the Sailor lineage whose structure however remains a little unclear. At last, the Copia elements remain rare and results from the consistent evolutionary success of the same three clades.

## Methods

### Animal collection, DNA and RNA extraction, sequencing, and assembly

Worms were collected in contrasted marine habitats from shallow-water/intertidal to deep-sea environments from different regions of the globe over the past 12 years which also includes extreme environments such as the cold Antarctic waters and the hot hydrothermal vents of the Pacific (see Additional file 12 for detail). Upon recovery, specimens were flash-frozen in liquid nitrogen and then transferred at − 80 °C in a deep freezer. Once back in the laboratory, total RNA extraction was performed for our whole set of 26 species using a Trizol/Chloroform protocol and a Retsch MM300 ball mill. Total RNA were re-precipitated after a PVPP (1%) treatment to eliminate polyphenols. Genomic DNA was also purified from one specimen per species for a subset of 14 species using a standard CTAB 2%/PVP 1% protocol [57]. Dry DNA pellets were suspended in DNAse-free water and the remaining contaminants were eliminated by adding 1% of PVPP. RNA-seq and DNA-seq libraries were produced at Genome Québec and sequenced accordingly on a HiSeq 2000 to obtain 150 bp paired-end reads using Illumina TruSeq kit for paired-end reads [58], following mRNA stranded purification and Covaris fragmentation, respectively. According to the species, the Illumina RNAseq sequencing effort varied from a quarter of a lane (40 million reads) to one full lane (160 million reads). Raw genomic data have been deposited in NCBI and are available under the Bioproject PRJNA766809.

For 15 species, random subsets of paired-end fastq reads were produced by down sampling a fraction of about 20, 40, 60, 80, 100, 120 and 140 millions of reads (seqtk sample -s100 readlibrary1.fq.gz 0.1 > subset1.fq). Transcriptomes were de novo assembled with the Trinity 2.8.4 software (https://github.com/trinityrnaseq/trinityrnaseq/releases/tag/Trinity-v2.8.4) where raw reads were subjected to a screening process using the trimming and normalization options following the bioinformatic parameters: Trinity --seqType fq --left X --right X --trimmomatic --quality_trimming_params “ILLUMINACLIP:illumina.fa:2:30:10 LEADING:5 TRAILING:5 SLIDINGWINDOW:4:15 MINLEN:36” --normalize_reads --max_memory 50G --CPU 8 --output trinity_dir_X.

### Genome size estimation by flow cytometry

Cell suspensions were prepared from a single individual using parietal muscle tissue. We had at our disposal only 1 specimen of *Branchinotogluma* sp.; otherwise, genome size estimates were based on measurements from at least 2 individuals of a species with multiple replicates. Samples of body tissues (10–50 mg) were finely chopped with a razor blade in 500 ml of Nuclei Isolation Buffer twice diluted (NIB/2, [59]) supplemented with final concentration of 0.1% polyvinylpyrrolidone (to immobilize phenolics), 0.1% RNase, 0.1% BSA and 0.2% Triton. Samples were compared against an internal standard of known genome size either Chicken Red Blood Cells (CRBC, 2C = 2.33 pg) or Human Blood Cells (HBC, 2C = 6.66 pg). Extracts were filtered through 50 μm nylon mesh and stained on ice with Propidium Iodide 30 ng/mL (final concentration). The samples were analyzed on an FACS Canto II (Becton Dickinson, San Jose, CA) equipped with a 488 nm laser and the standard filter setup. Results, given as C-values, are deduced from 2C nuclei of individuals considered diploid. The haploid nuclear DNA content is expressed in picograms or million base pairs, where 1 pg = 978 Mbp [60]. For two of the species we do not have the biological material necessary for the measurements. In the case of *Harmothoe* sp., we have therefore chosen to use the average of the estimates obtained for the other two *Harmothoe* (2.65 Gb). In the case of *Levensteiniella plicata* we have chosen to use the average of the estimates obtained on the two *Lepidonotopodium* considering that these three species are grouped in the classification (1.78Gb).

### Detection of TE sequences in genomes

Estimation of the abundance and the respective proportion of each LTR-retrotransposon family using reads were carried out using the DnaPipeTE software with default parameters [37]. TE abundance have been calculated as the read fraction corresponding to 1X genome coverage that align against each TE consensuses. For each species, reads that map on the corresponding mitochondrial genomes using the BWA software [61] were first discarded. DnaPipeTE were run on read subsamples ranging between coverage of 0.01x and 0.5x in intervals of 0.05x (11 runs). For each of the 11 runs per species, we selected the subsample yielding the highest contig N50 in the assembly step of dnaPipeTE, as a measure of optimized read subsampling. TE families were annotated using BLAST against RepBase 10/10/2017 version [62]. In a second step, and for comparison purposes, the consensus genomic sequences of the transposable elements were re-annotated using the same pipeline as the one used for the TE sequences in transcriptomes (see above).

### Detection of TE sequences in transcriptomes

A python script was written to optimize the detection of TEs in the newly assembled transcriptomes, which includes 5 major steps (http://gofile.me/2ppPR/sY5fRoTUA):

(1) Transcripts that possess a putative TE sequence were detected by BLASTX similarity-search on a custom database, LAC28, based on the 18,011 amino-acid sequences of the Repeatpeps library (Nov 2018, http://repeatmasker.org/libraries/) appended with published RT/RnaseH sequences from mollusk BEL/Pao elements and from various GalEa retrotransposons [21, 27]. As the library contains only a few elements from annelids, we also enriched our database with 410 annelid sequences newly identified. For this purpose, we manage to represent all types of TEs with between 3 to 19 sequences mainly coming from six species (three Alvinellidae and three Polynoidae, see Additional file 13 for details and Additional file 14 for amino acid sequences). These sequences, previously detected by BLASTX searches, were manually checked to fully correspond to TEs by comparison using CENSOR on Repbase (https://www.girinst.org/). Moreover, in a preliminary test, we also used CENSOR to confirm 5873 putative TE sequences revealed by the first version of our database (between 22 to 286 sequences for each TE type). This allows us to discard from our LAC28 database sequences giving unreliable results; so that thereafter more than 95% of the sequences revealed by BLASTX searches actually correspond to an element of the expected type.

(2) BLASTX results were filtered to eliminate a maximum of false positives. Indeed, according to the earlier experience, analyses of 200 putative TE sequences reveal that most hits which have an identity match < 25%, an alignment length < 50, a number of gap > 15, or an e-value > e^− 22^ do not correspond in fact to identifiable TEs.

(3) Remaining transcripts were then checked by cross-matching using a tBLASTn search of TE sequences of LAC28 on a database corresponding to selected transcripts that possess a putative TE sequence. Transcripts that were not recovered or that did not correspond to the same element type in both BLASTX and tBLASTn searches were discarded.

(4) All transcripts that passed the filters and possess a TE sequence were grouped into different fasta files according to the type of element. Here we use the term superfamily/type since none of these terms is universally accepted. Overall, we considered 40 types of elements in annelids (20 DNA transposons including Helitron elements, 14 LINEs, Penelope elements, and 5 retrotransposons), which correspond to the usual groups defined in RepeatMasker and Repbase libraries (see Additional file 15 for details). All transcripts that potentially contain a TE fragments are provided in Additional file 16.

(5) For each type of element, the number of families was then estimated. Output of Trinity Assembly encoded different grouping levels in the Trinity fasta accession with ‘isoform’, ‘gene’ and ‘cluster’. For example, the accession ‘TRINITY_DN1000_c115_g5_i1’ indicates Trinity read cluster ‘TRINITY_DN1000_c115’, gene ‘g5’, and isoform ‘i1’. It seems logical to assume that repeated coding sequences in the genome are grouped at the cluster level. To verify this, we accurately compared the sequences of 102 transcripts of Copia elements from six different species, thus representing 102 isoforms, 81 genes and 63 clusters. In all cases, sequences of isoforms of the same gene were very similar (> 95% identity) and thus belong to the same family. In most cases, the sequences of the genes of a same cluster were sufficiently close (> 80% identity) to be considered as representing the same family of elements. In only 3 cases we could note a discrepancy between the estimated number of families based on the sequence identity and the number of clusters established by Trinity. Twice, Trinity grouped two sequences that were not manually alignable. Conversely, once Trinity separated two transcripts into two different clusters whereas the sequences belonged to the same element (difference due to the presence of a large deletion in one of them). In conclusion, the sequences of the different isoforms and genes defined by Trinity were relatively close; while those of the clusters were easily distinguishable (i.e. the reads resulting from the different transcribed copies of the same family are assembled within the same cluster). As a consequence, we have considered that for the TEs each cluster resulting from the assembly represented a distinct family (= an element).

### Cladistic analyses

Phylogenetic analyses were performed as in [21] on amino acid sequences corresponding to the RT/RNaseH or Integrase domains of the newly characterised sequences, reference elements from Repbase or GypsyDatabase, and previously identified Copia and BEL/Pao retrotransposons. Boundaries of RT/RNaseH domains have been determined by BLASTX searches according to those defined for RT 5′ part and RNaseH 3′ part of Copia, BEL/Pao and Gypsy multiple alignments defined in the GypsyDatabase. DNA sequences were translated using a custom-made script and the longest representative of each family was selected.

Multiple alignments of protein sequences were performed using MAFFT [63] and are freely available at http://gofile.me/2ppPR/1XcV54vF2. After a manual curation of the alignments, phylogenetic analyses were conducted using Neighbor Joining [64] and the pairwise deletion option of the MEGA5.2 software [65]. Using the Topali2.3 software [66], the best-fitted substitution model retained was the JTT model [67] with a gamma distribution. Support for individual groups was evaluated with non-parametric bootstrapping [68] using 100 replicates.

### Detection of Argonaute sequences in transcriptomes

Argonaut protein sequences were searched in the transcriptomes by BLASTX (e-value < e^− 70^) using as query 10 reference sequences from *Capitella teleta* (Piwi1_Ct ELT87139, Piwi2_Ct ELU02261), *Drosophila melanogaster* (AUB_Dm AGA18939, Piwi_Dm AAD08705, AGO1_Dm NP_725341.1, AGO2_Dm NP_648775, AGO3_Dm ABO27430), *Trypanosoma brucei* (Piwi-like_Tb AAR10811, argonaute-like1_Tb AAR10810, and *Arabidopsis thaliana* AGO_AT CAA0278680 (some of them also include in the phylogeny). Sequences were then translated and, if long enough, the largest of each transcript cluster was included in a phylogenic tree following the method previously used for TEs and including also the reference proteins Piwi1_Av KM406471, Piwi2_Av KM406472, AUB_Bm NP_001098066, AGO3_Bm NP_001098067, Piwi1_Dr NP_899181, Piwi2_Dr ACF35261, AGO1_Mm NP_700452, Piwi1_Mm NP_067286, Piwi2_Mm NP_067283, Piwi1_Nv XP_001641994. Annelid protein sequences are freely available at http://gofile.me/2ppPR/Rvs88Fkpy.

To search for putative RNA-cluster fragments, a ‘force translated search’ was performed using the CENSOR software (Repbase https://www.girinst.org/) on the TE transcripts of more than 8 kb of the 26 transcriptomes in order to analyze their TEs diversity. All outputs were then manually checked to isolate those containing at least 8 fragments of different elements belonging to at least 3 different classes.

## Supplementary Information

**Additional file 1.** Summary of previous data on annelid transposable elements (.pdf)

**Additional file 2.** Standard classification of annelid species studied. (.pdf)

**Additional file 3.** DNA C-values for species of annelids. (.xls)

**Additional file 4.** Scatter plot showing the relationship between transcripts, clusters and TE family numbers. The graphs represent the data obtained on all the assembled transcriptomes for the 15 species of annelids (all), as well as the detail for the transcriptomes obtained for increasing subsamples of reads (Millions). (.pdf)

**Additional file 5.** Heat maps of TE types identified in 12 remaining annelids according to the number of reads used for the transcriptome assembly. (.xls)

**Additional file 6. **Heat maps of TE types identified in the low-coverage genome or in the transcriptome of the six remaining annelids. For each species, proportions of TEs in the genome (G, left column) or transcriptomes assembled from 40 million reads (T, right column) are shown the same way as in Figure 3. The total number of TE families detected is indicated above each column. Brasp - *Branchinotogluma* sp*.*; Lepwil - *Lepidonotopodium williamsae*; Harful - *Harmothoe fuligineum*; Harsp - *Harmothoe* sp*.*; Pefu - *Pettitbonesia furcosetosa*; Pgras - *P.grasslei.* (.xls)

**Additional file 7.** Phylogenetic relationships of Integrase sequences of Copia retrotransposons based on Neighbor-Joining analysis of Integrase domain amino acid sequences. The Copia families from annelids are indicated in color. Node statistical support values (>70 %) come from non-parametric bootstrapping using 100 replicates. (.pdf)

**Additional file 8.** Phylogenetic relationships of Integrase sequences of BEL/Pao retrotransposons based on Neighbor-Joining analysis of Integrase domain amino acid sequences. The BEL/Pao families from annelids are indicated in color. Node statistical support values (>70 %) come from non-parametric bootstrapping using 100 replicates. (.pdf)

**Additional file 9.** Phylogenetic relationships among Gypsy clades. This tree is a simplified representation of Figure 9, in which annelid elements from the same clade are represented by compressed subtrees. All LTR-retrotransposons from a clade found in annelids are depicted in color. The reference Gypsy elements and Gypsy clades previously reported in the GypsyDatabase are in black. Node statistical support (>70%) was obtained through non-parametric bootstrapping using 100 replicates. (.pdf)

**Additional file 10.** Distribution of Gypsy clades within annelids. Tanglegram-like representation of connections between Gypsy clades and annelid species within an ordered list of species names according to their phylogenetic relationships. (.pdf)

**Additional file 11.** Example of long transcripts containing multiple fragments of diverse transposable element origins reminiscent of piRNA cluster structure. Schematic representations of transcripts (> 8 kb) identified six annelid transcriptomes were obtained using the CENSOR software from Repbase. For each sequence, the upper scheme represents the portion of transcript corresponding to TEs (red). The scheme below corresponds to the annotation of TE fragments (DNA transposons in purple, Non-LTR retrotransposons in blue, LTR-retrotransposon (improperly including the YR elements) in grey, simple repeat in green). Details of annotated fragments are indicated in the associated tables. (.xls)

**Additional file 12.** Annelid species localization. (.xls)

**Additional file 13.** Source of annelid elements added to the reference database. (.xls)

**Additional file 14. **Amino acid sequences of annelid elements added to the reference database. (.fas)

**Additional file 15.** Categories of transposable elements used and their correspondence in the referenced databases. (.xls)

**Additional file 16. **Annelid transcripts that potentially contain a TE fragment (.fas)

## Data Availability

All data generated or analysed during this study are included in this published article (and its supplementary information files). All the datasets used and analyzed during the current study are available from the corresponding authors on reasonable request.
