# Supplementary material for: Comparative genomic and transcriptomic analyses of transposable elements in polychaetous annelids highlight LTR retrotransposon diversity and evolution
Source: Mob DNA. 2021 Oct 29;12:24. doi: 10.1186/s13100-021-00252-0 (PMC8556966; doi:10.1186/s13100-021-00252-0)
Supplement: Supplementary file 1 — Additional file 1. Summary of previous data on annelid transposable elements (.pdf) [file 13100_2021_252_MOESM1_ESM.pdf]

What was previously known about TE in Annelids?

Restricted informations were available concerning Annelids TE contents.

The first source of information was a brief description of TEs within few sequenced genomes distributed among the three major annelid groups: the earthworm *Eisenia fetida* [1], the leeches *Hirudinaria manillensis* [2] and *Helobdella robusta* [3], and the marine polychaetes *Capitella teleta* [3], *Lamellibrachia luymesi* [4]. In the earthworm, more than one million copies of TEs were detected, a majority of which were transposons (43%), followed by LTR-retrotransposons (31%) and LINEs (18%). In *H. manillensis* more than 14,000 families have been found with RepeatModeler. An equivalent proportion of transposons (4118 families, with a large majority of Crypton, TcMariner and hAT) and LINEs (4308, mostly L2, as well as R2, I and CR1) were observed. The LTR-retrotransposon families are slightly numerous with a very large number of Gypsy (5246) and few Copia (446) and BEL/Pao (69). Considering only families with more than 10 copies, only 1901 "Repeat element families" were detected with RepeatScout in *H. robusta* (16.87% of the genome) and 5220 in *C. teleta* (8.58% of the genome). These two species show a very large majority of LINEs with 69% and 57% of the copies, respectively (mainly CR1, RTE, CRE and R2 in *H. robusta,* Proto and Crack in *C. teleta*); 10% of transposons (with a large majority of MULEs (Rehavkus) and hAT in *H. robusta*, and a majority of TC1/mariner and Maverick (polinton) in *C. teleta*). The amount of LTR was more variable as they were rare in the leech (less than 2%) and abundant in polychaetes (12%) with Gypsy and no or few BEL/Pao. In *L. luymesi*, the combination of RepeatModeler and RepeatMasker reveals that 35 % of the genome is made up of interspersed repeats, the majority of which are DNA elements and LINEs (14.81 % and 13.99 % of the genome coverage) and much less LTR elements (2.52 %).

The second source of information comes from articles dedicated to a particular element or superfamily of elements, in which annelids can be cited among many other taxa. Most of these studies referred to transposons: a TcMariner transposon, *EamaT1,* was characterized in the earthworm *Eisenia andrei* [5]; the presence in few annelids of MULEs of the groups of Phantom [6], MULE-Trichinella and Rehavkus [7], and Academ [8] were confirmed, as well as Maveriks element in *Platynereis dumerilii* [9]. Regarding retrotransposons, an RTE element was detected in *Lumbricus terrestris* [10]; annelid Daphne elements were included in the analysis of diverse LINEs [11]; and two Dirs1-like elements were characterized in *Capitella sp* [12].

1. Paul S, Arumugaperumal A, Rathy R, Ponesakki V, Arunachalam P, Sivasubramaniam S. Data on genome annotation and analysis of earthworm Eisenia fetida. Data Brief. 2018;20:525–34.

2. Guan D-L, Yang J, Liu Y-K, Li Y, Mi D, Ma L-B, et al. Draft Genome of the Asian Buffalo Leech Hirudinaria manillensis. Front Genet. 2020;10. doi:10.3389/fgene.2019.01321.

3. Simakov O, Marletaz F, Cho S-J, Edsinger-Gonzales E, Havlak P, Hellsten U, et al. Insights into bilaterian evolution from three spiralian genomes. Nature. 2013;493:526–31.

4. Li Y, Tassia MG, Waits DS, Bogantes VE, David KT, Halanych KM. Genomic adaptations to chemosymbiosis in the deep-sea seep-dwelling tubeworm Lamellibrachia luymesi. BMC Biology. 2019;17:91.

5. Jee SH, Kim GE, Hong SH, Seo SB, Shim JK, Park SC, et al. Characterization of EamaT1, a member of maT family of transposable elements from the earthworm Eisenia andrei (Annelida, Oligochaeta). Mol Genet Genomics. 2007;278:479–86.

6. Marquez CP, Pritham EJ. Phantom, a new subclass of Mutator DNA transposons found in insect viruses and widely distributed in animals. Genetics. 2010;185:1507–17.

7. Dupeyron M, Singh KS, Bass C, Hayward A. Evolution of Mutator transposable elements across eukaryotic diversity. Mob DNA. 2019;10:12.

8. Kojima KK. AcademH, a lineage of Academ DNA transposons encoding helicase found in animals and fungi. Mob DNA. 2020;11:15.

9. Pritham EJ, Putliwala T, Feschotte C. Mavericks, a novel class of giant transposable elements widespread in eukaryotes and related to DNA viruses. Gene. 2007;390:3–17.

10. Zupunski V, Gubensek F, Kordis D. Evolutionary dynamics and evolutionary history in the RTE clade of non-LTR retrotransposons. Mol Biol Evol. 2001;18:1849–63.

11. Metcalfe CJ, Casane D. Modular organization and reticulate evolution of the ORF1 of Jockey superfamily transposable elements. Mob DNA. 2014;5:19.

12. Piednoël M, Gonçalves IR, Higuet D, Bonnivard E. Eukaryote DIRS1-like retrotransposons: an overview. BMC Genomics. 2011;12:621.
