# Supplementary figures and images for: Comparative genomic and transcriptomic analyses of transposable elements in polychaetous annelids highlight LTR retrotransposon diversity and evolution
Source: Mob DNA. 2021 Oct 29;12:24. doi: 10.1186/s13100-021-00252-0 (PMC8556966; doi:10.1186/s13100-021-00252-0)

Order PHYLLODOCIDA

Family POLYNOIDAE

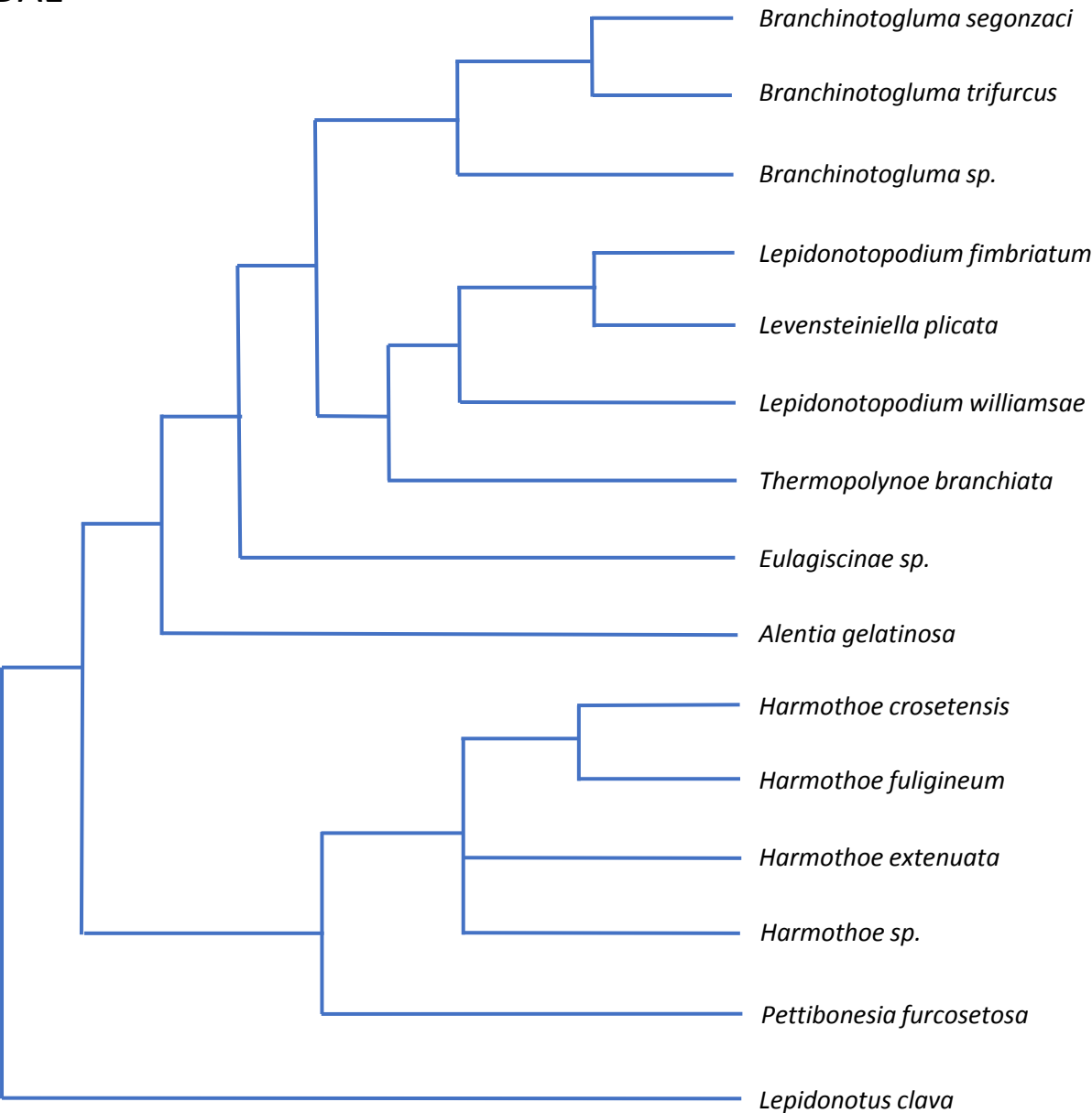

Order TERESELLIDA

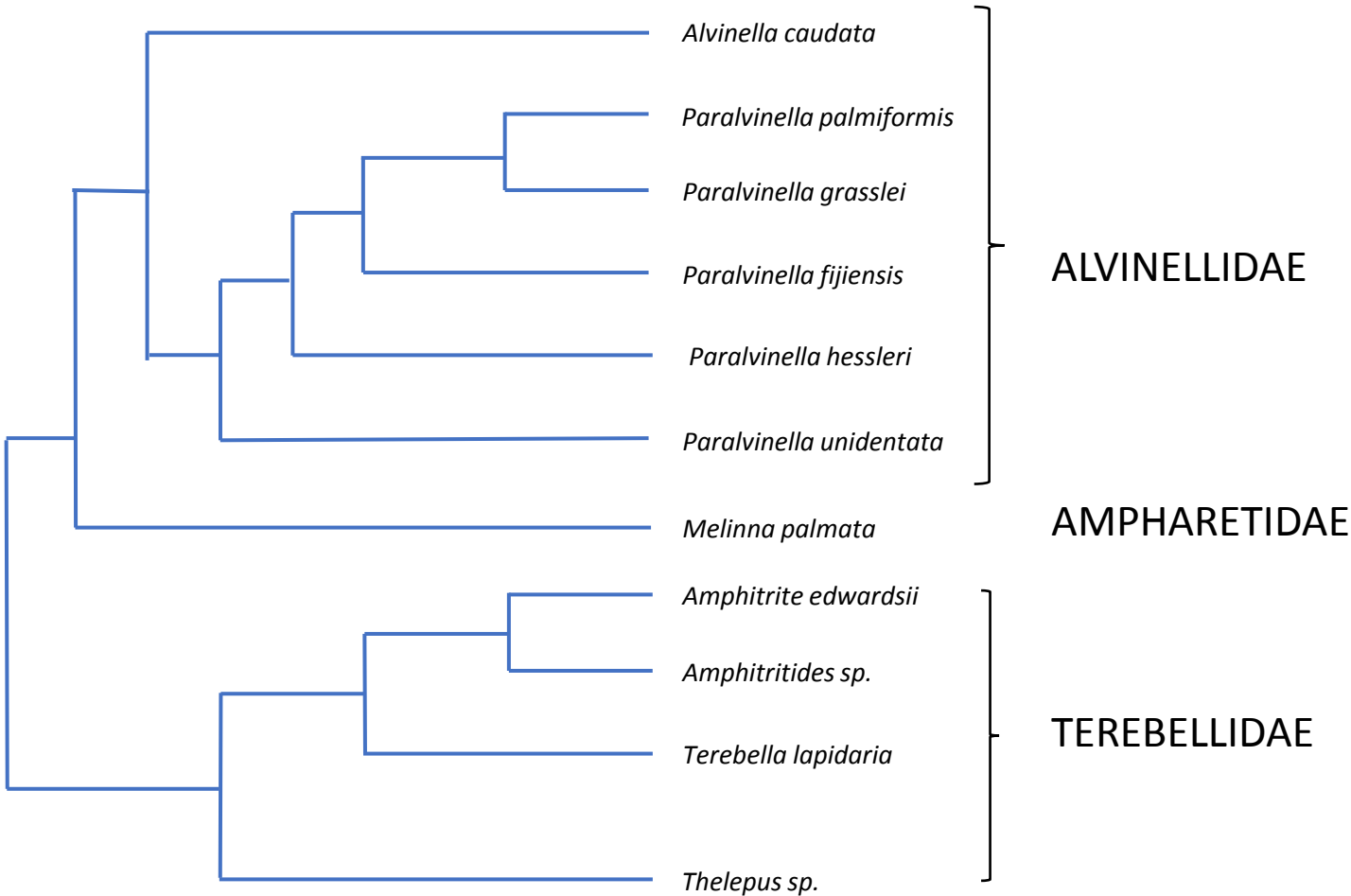

Supplement: Supplementary file 2 — Additional file 2. Standard classification of annelid species studied. (.pdf) [file 13100_2021_252_MOESM2_ESM.pdf]

GalEa

Hydra

Comol

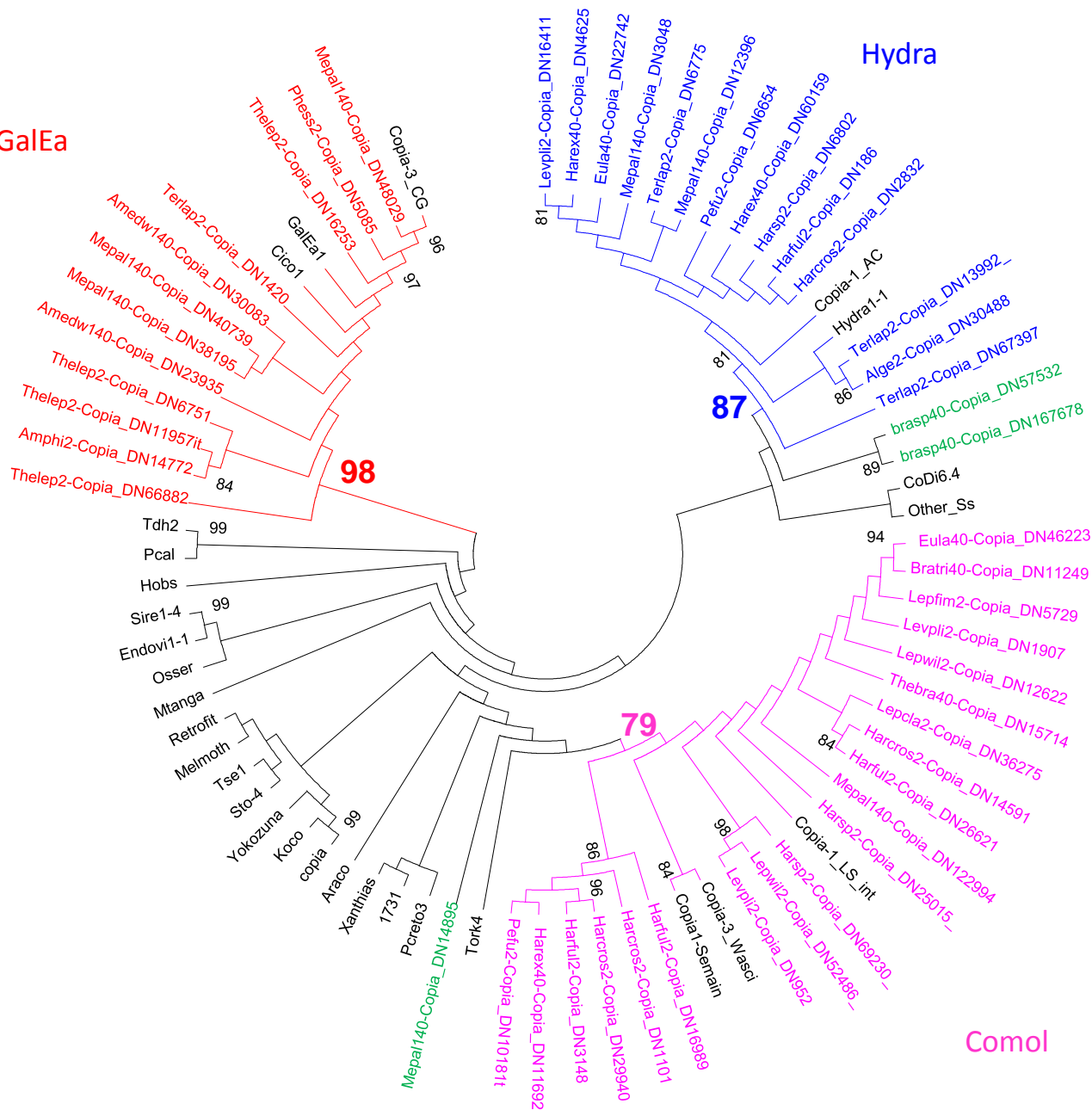

Supplement: Supplementary file 7 — Additional file 7. Phylogenetic relationships of Integrase sequences of Copia retrotransposons based on Neighbor-Joining analysis of Integrase domain amino acid sequences. The Copia families from annelids are indicated in color. Node statistical support values (>70 %) come from non-parametric bootstrapping using 100 replicates. (.pdf) [file 13100_2021_252_MOESM7_ESM.pdf]

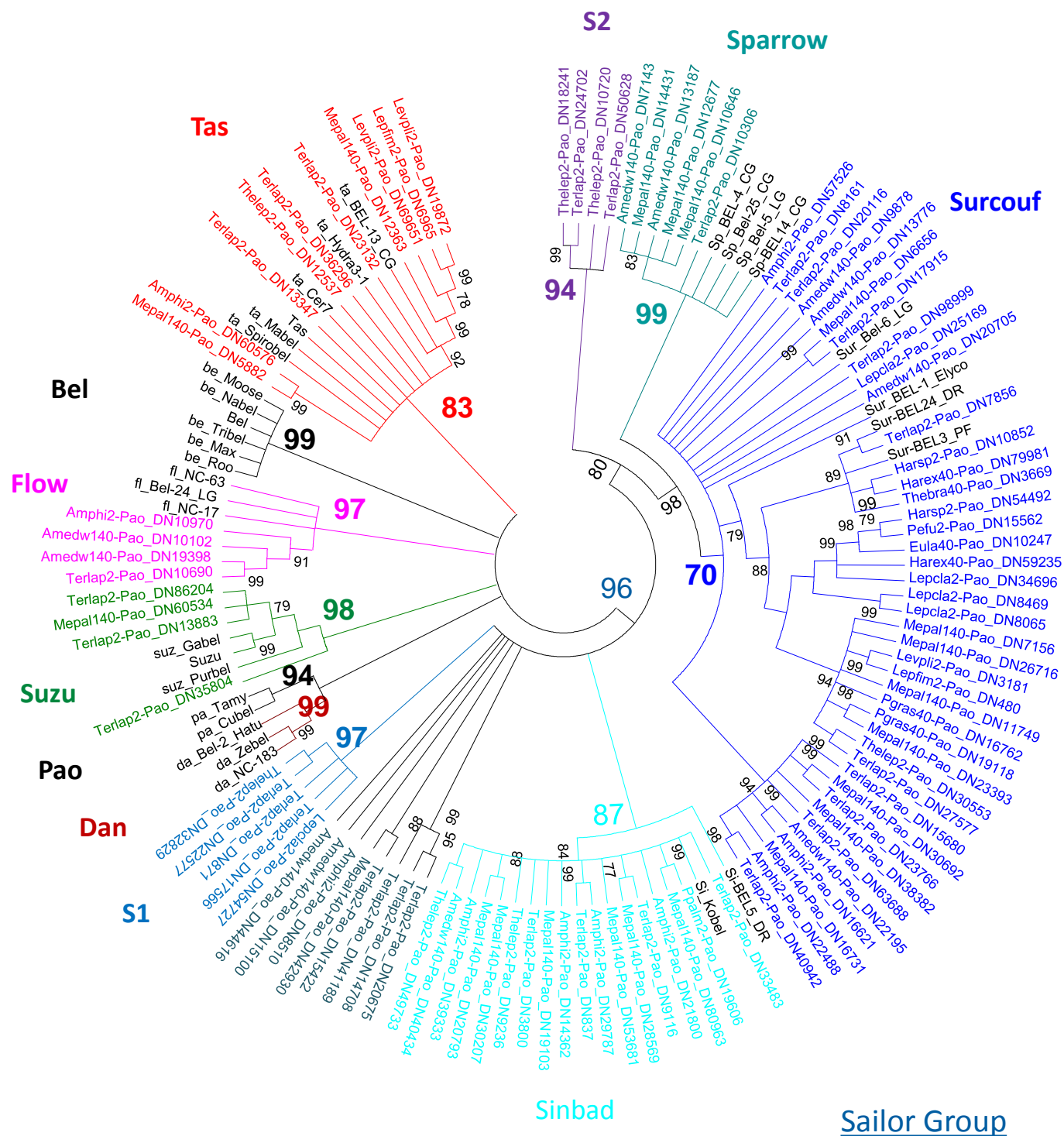

Supplement: Supplementary file 8 — Additional file 8. Phylogenetic relationships of Integrase sequences of BEL/Pao retrotransposons based on Neighbor-Joining analysis of Integrase domain amino acid sequences. The BEL/Pao families from annelids are indicated in color. Node statistical support values (>70 %) come from non-parametric bootstrapping using 100 replicates. (.pdf) [file 13100_2021_252_MOESM8_ESM.pdf]

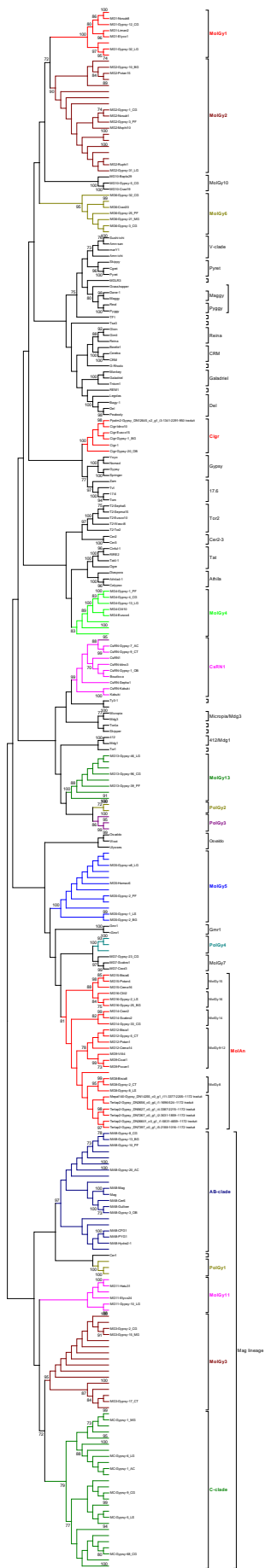

Supplement: Supplementary file 9 — Additional file 9. Phylogenetic relationships among Gypsy clades. This tree is a simplified representation of Figure 9, in which annelid elements from the same clade are represented by compressed subtrees. All LTR-retrotransposons from a clade found in annelids are depicted in color. The reference Gypsy elements and Gypsy clades previously reported in the GypsyDatabase are in black. Node statistical support (>70%) was obtained through non-parametric bootstrapping using 100 replicates. (.pdf) [file 13100_2021_252_MOESM9_ESM.pdf]

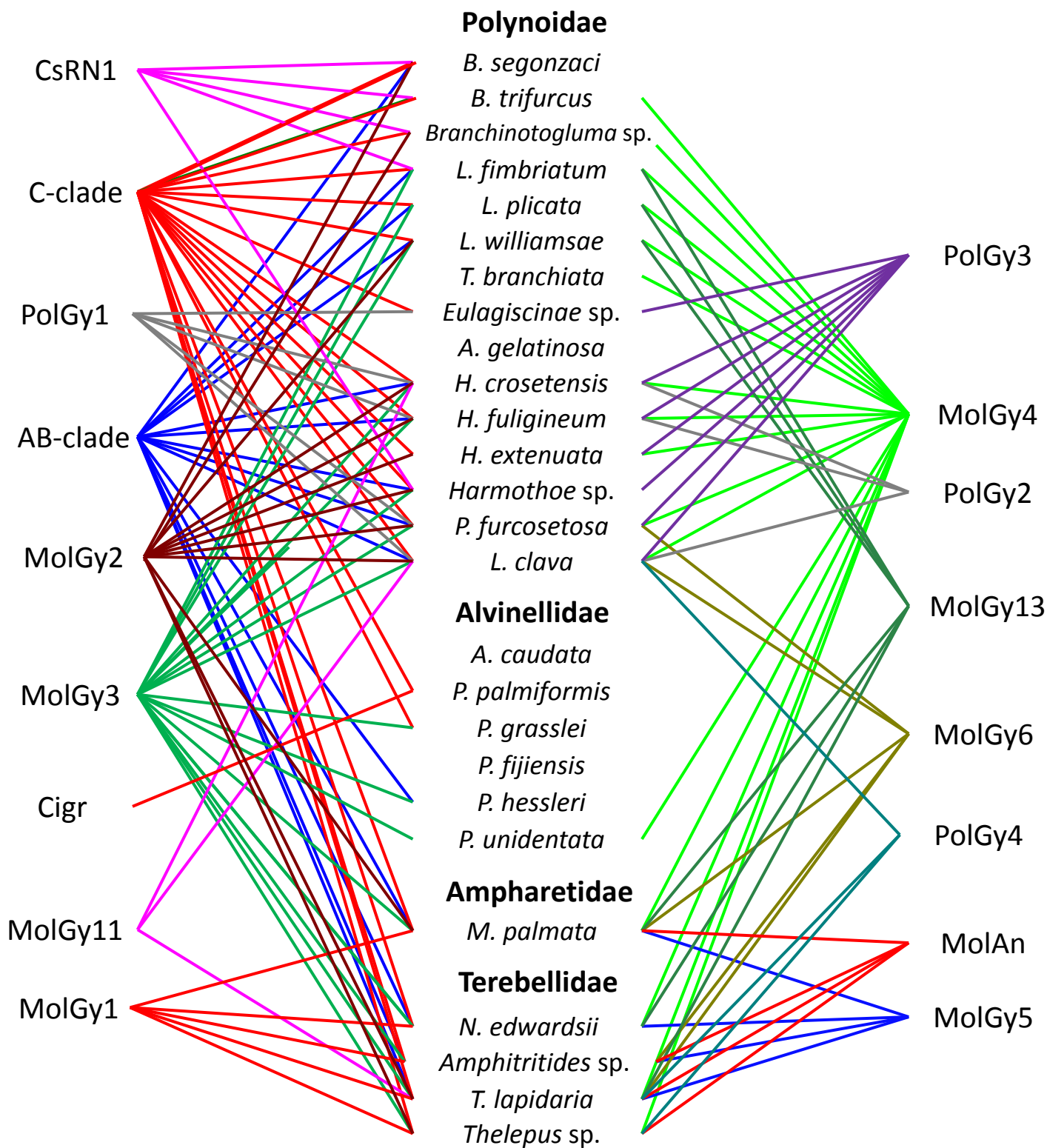

Supplement: Supplementary file 10 — Additional file 10. Distribution of Gypsy clades within annelids. Tanglegram-like representation of connections between Gypsy clades and annelid species within an ordered list of species names according to their phylogenetic relationships. (.pdf) [file 13100_2021_252_MOESM10_ESM.pdf]
