# Supplementary material for: Comparative genomic and transcriptomic analyses of transposable elements in polychaetous annelids highlight LTR retrotransposon diversity and evolution
Source: Mob DNA. 2021 Oct 29;12:24. doi: 10.1186/s13100-021-00252-0 (PMC8556966; doi:10.1186/s13100-021-00252-0)

Clusters *versus* Transcripts

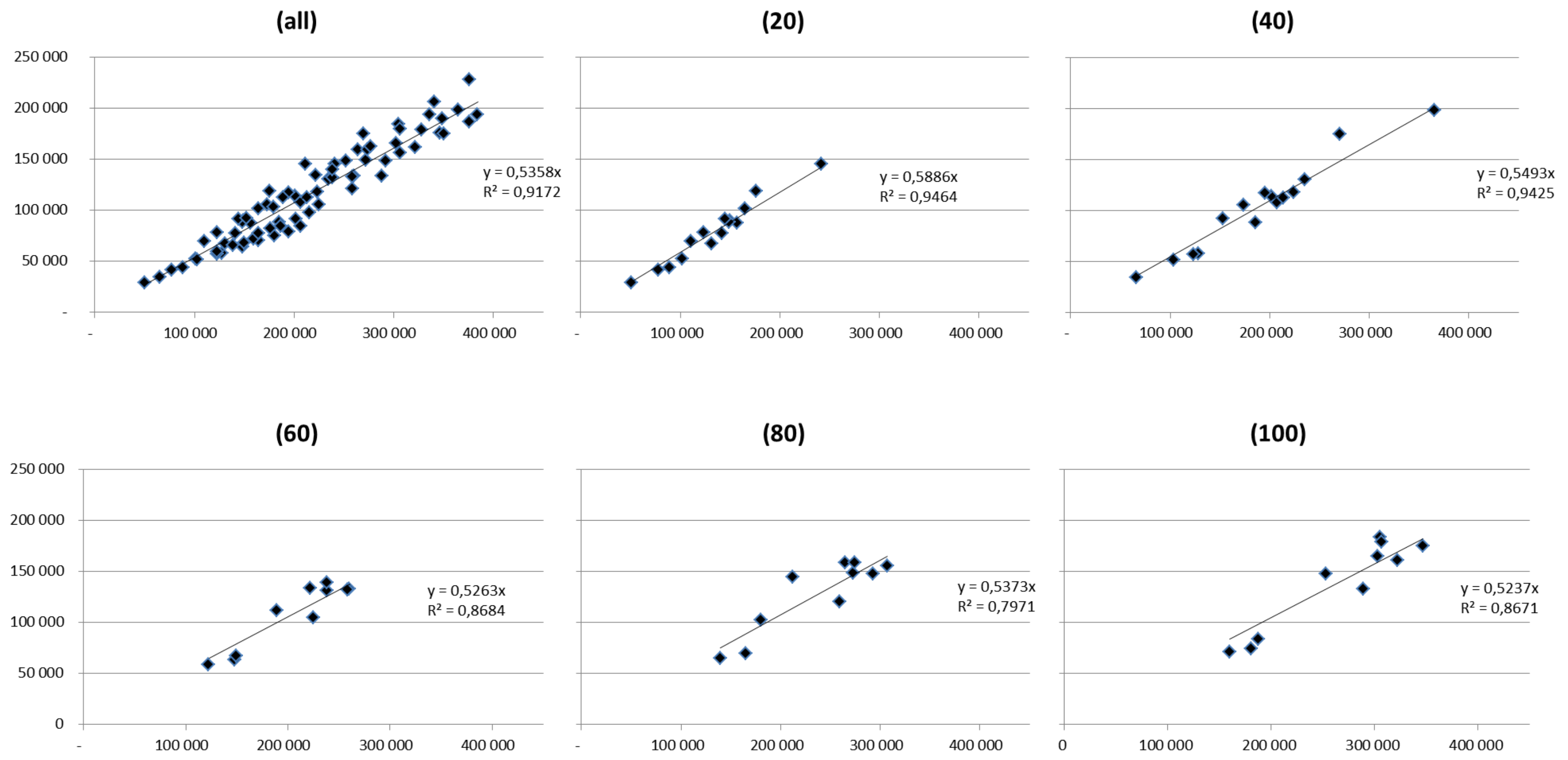

# TE Families *versus* Transcripts

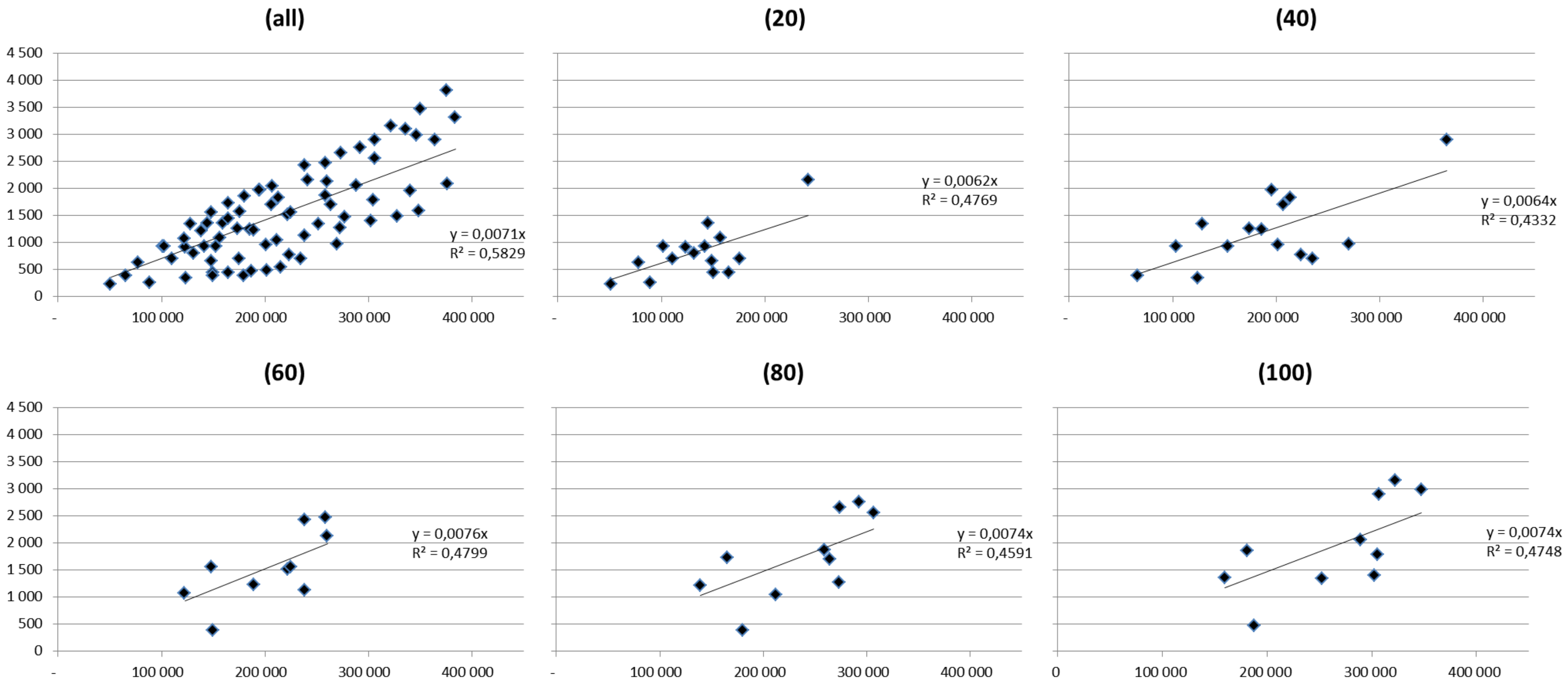

# TE Families *versus* Clusters

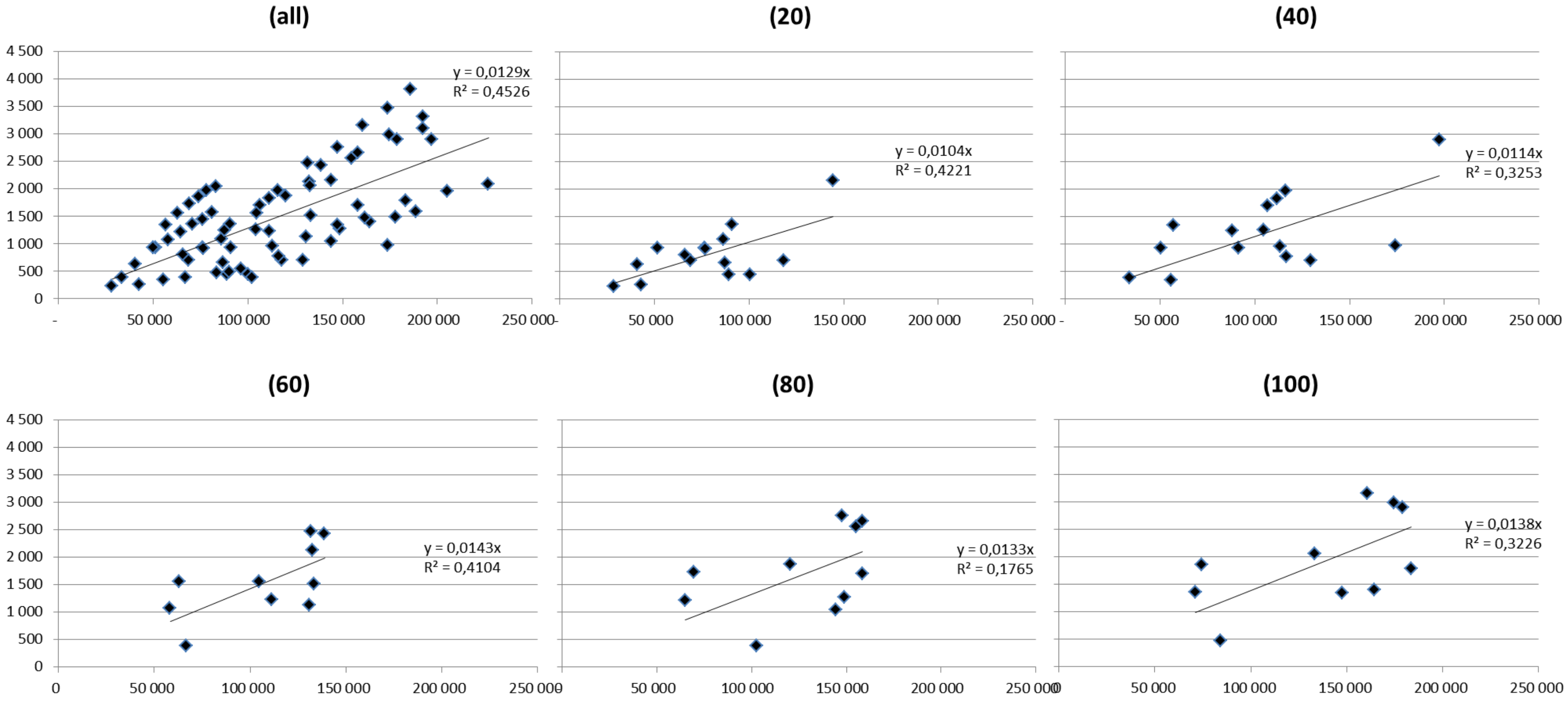

Supplement: Supplementary file 4 — Additional file 4. Scatter plot showing the relationship between transcripts, clusters and TE family numbers. The graphs represent the data obtained on all the assembled transcriptomes for the 15 species of annelids (all), as well as the detail for the transcriptomes obtained for increasing subsamples of reads (Millions). (.pdf) [file 13100_2021_252_MOESM4_ESM.pdf]
