# Supplementary material for: Comparative genomic and transcriptomic analyses of transposable elements in polychaetous annelids highlight LTR retrotransposon diversity and evolution
Source: Mob DNA. 2021 Oct 29;12:24. doi: 10.1186/s13100-021-00252-0 (PMC8556966; doi:10.1186/s13100-021-00252-0)
Supplement: Supplementary file 6 — Additional file 6. Heat maps of TE types identified in the low-coverage genome or in the transcriptome of the six remaining annelids. For each species, proportions of TEs in the genome (G, left column) or transcriptomes assembled from 40 million reads (T, right column) are shown the same way as in Figure 3. The total number of TE families detected is indicated above each column. Brasp - Branchinotogluma sp.; Lepwil - Lepidonotopodium williamsae; Harful - Harmothoe fuligineum; Harsp - Harmothoe sp.; Pefu - Pettitbonesia furcosetosa; Pgras - P.grasslei. (.xls) [file 13100_2021_252_MOESM6_ESM.pdf]

	Brasp		Lepwil		Harful		Harsp		Pefu		Pgras	
	G	T	G	T	G	T	G	T	G	T	G	T
Total number	1679	2903	833	1260	1484	1699	1574	1830	779	1979	323	395
**Transposon**												
Academ	1,1%	0,8%	3,6%	3,0%	2,8%	2,5%	0,3%	2,6%	1,2%	2,1%	2,5%	2,5%
Crypton	0,1%	0,2%	0,4%	0,9%	1,8%	2,4%	0,6%	1,5%	0,3%	2,1%	0,0%	0,8%
Dada	0,0%	0,1%	0,0%	0,2%	0,0%	0,0%	0,0%	0,0%	0,0%	0,0%	0,3%	1,3%
CMC	0,1%	0,1%	0,4%	0,5%	0,0%	0,2%	0,1%	0,0%	0,0%	0,2%	0,0%	1,3%
Ginger	0,2%	0,3%	0,1%	0,6%	0,1%	0,1%	0,2%	0,1%	0,0%	0,1%	0,0%	1,5%
Harbinger	0,7%	1,3%	0,1%	0,2%	2,4%	3,2%	1,2%	3,4%	1,2%	2,6%	0,0%	1,0%
hAT	0,7%	0,5%	3,2%	4,7%	0,0%	0,8%	0,1%	0,3%	0,1%	0,5%	4,3%	5,1%
IS3EU	0,0%	0,0%	0,0%	0,2%	0,2%	0,9%	0,0%	0,8%	0,0%	0,4%	0,0%	0,0%
ISL2EU	0,0%	0,4%	0,8%	2,1%	0,1%	1,1%	0,2%	2,1%	0,0%	1,5%	3,7%	8,4%
Kolobok	0,2%	0,4%	0,2%	0,2%	0,1%	1,3%	0,1%	0,8%	0,0%	0,7%	0,9%	0,8%
Maverick	1,8%	1,0%	1,2%	1,2%	4,4%	1,6%	12,6%	2,6%	9,6%	3,7%	0,3%	0,0%
Merlin	0,0%	0,0%	0,4%	0,1%	0,3%	0,2%	0,3%	0,3%	1,0%	0,7%	0,0%	0,0%
MULE	0,0%	0,1%	1,3%	0,3%	0,1%	0,5%	0,1%	0,1%	0,0%	0,4%	1,5%	7,8%
P	0,0%	0,0%	0,5%	1,0%	0,0%	0,7%	0,2%	0,7%	0,0%	1,4%	0,3%	1,0%
PiggyBac	0,0%	0,0%	0,0%	0,0%	0,4%	0,3%	0,3%	0,4%	0,0%	0,2%	0,3%	0,3%
Sola	0,0%	0,0%	1,0%	1,6%	3,0%	2,1%	1,2%	1,5%	2,6%	1,6%	0,0%	1,3%
TcMariner	0,1%	0,8%	1,8%	2,9%	0,4%	2,1%	0,1%	0,8%	0,5%	0,9%	2,5%	6,1%
Zator	0,0%	0,0%	0,0%	0,1%	1,0%	0,6%	0,1%	0,9%	0,0%	0,2%	0,0%	0,0%
Zisupton	0,1%	0,1%	0,0%	0,1%	0,0%	0,2%	0,0%	0,4%	0,0%	0,3%	0,0%	0,3%
Helitron	0,0%	0,9%	0,0%	1,6%	1,2%	1,7%	0,7%	1,2%	0,8%	2,4%	0,0%	4,3%
**LINE**												
CR1	0,1%	0,1%	2,3%	2,5%	1,6%	1,5%	1,5%	2,6%	0,8%	2,1%	11,5%	8,4%
I	0,7%	0,5%	0,2%	0,3%	0,5%	0,3%	0,3%	0,4%	0,5%	0,5%	7,1%	6,6%
L1	0,2%	0,4%	0,5%	0,2%	0,9%	0,3%	0,4%	0,9%	1,9%	0,7%	4,6%	0,8%
Nimba	0,6%	0,7%	4,8%	3,5%	0,3%	0,0%	0,4%	0,4%	2,2%	1,0%	7,1%	3,0%
L2	0,0%	0,0%	0,6%	0,3%	0,5%	0,7%	1,7%	1,4%	0,3%	0,4%	13,9%	8,9%
L2A	0,0%	0,0%	0,0%	0,0%	0,0%	0,3%	0,1%	0,2%	0,0%	0,4%	1,2%	0,3%
L2B	0,0%	0,0%	1,2%	1,4%	3,8%	5,1%	4,4%	6,8%	1,5%	5,6%	0,0%	0,0%
Crack	50,0%	42,4%	32,3%	25,2%	20,4%	28,6%	23,8%	27,2%	8,2%	22,2%	0,6%	0,5%
Daphne	0,0%	0,0%	0,6%	0,6%	8,8%	9,2%	11,3%	8,7%	3,2%	7,8%	5,9%	2,3%
Proto2	0,0%	0,0%	0,6%	0,6%	0,3%	0,5%	0,0%	0,4%	0,6%	0,7%	5,3%	3,0%
R2	0,0%	0,1%	0,0%	0,2%	0,0%	0,1%	0,1%	0,1%	0,0%	0,1%	0,3%	1,0%
Rex	0,0%	0,0%	0,1%	0,0%	0,0%	0,4%	0,0%	0,0%	0,0%	0,0%	0,0%	0,0%
RTE	0,0%	0,0%	0,7%	0,9%	0,3%	0,7%	0,2%	0,2%	1,0%	0,5%	2,2%	2,5%
RTEX	0,0%	0,0%	1,2%	0,0%	1,3%	1,6%	0,9%	1,0%	3,1%	2,7%	0,9%	0,8%
**Penelope**	0,0%	0,0%	0,0%	0,0%	4,7%	3,4%	7,1%	2,6%	0,9%	2,1%	0,9%	0,5%
**LTR-retrotransposons**												
Pao	0,0%	0,0%	0,0%	0,0%	0,0%	0,0%	0,1%	0,7%	1,2%	0,5%	2,2%	3,8%
Copia	0,0%	0,4%	0,2%	0,9%	1,3%	0,9%	0,9%	0,7%	1,7%	0,8%	0,0%	0,0%
Gypsy	13,2%	13,5%	28,9%	27,3%	27,6%	12,8%	19,8%	11,9%	49,0%	17,9%	17,6%	13,7%
**YR-retrotransposons**												
DIRS	13,2%	16,5%	1,1%	1,5%	2,1%	3,3%	3,4%	7,0%	2,6%	5,4%	0,0%	0,5%
Ngaro	17,0%	18,2%	9,6%	13,3%	7,4%	7,7%	5,5%	6,4%	4,1%	7,2%	1,9%	0,0%

0.0%
5.0%
10.0%
15.0%
20.0%
25.0%
30.0%
35.0%
40.0%
45.0%
50.0%
55.0%
60.0%
